# Comprehensive functional characterization of cancer–testis antigens defines obligate participation in multiple hallmarks of cancer

**DOI:** 10.1038/ncomms9840

**Published:** 2015-11-16

**Authors:** Kimberly E. Maxfield, Patrick J. Taus, Kathleen Corcoran, Joshua Wooten, Jennifer Macion, Yunyun Zhou, Mark Borromeo, Rahul K. Kollipara, Jingsheng Yan, Yang Xie, Xian-Jin Xie, Angelique W. Whitehurst

**Affiliations:** 1 Simmons Comprehensive Cancer Center, UT-Southwestern Medical Center, Dallas, Texas 75390, USA; 2 Department of Pharmacology, University of North Carolina at Chapel Hill, Chapel Hill, North Carolina 27599, USA; 3 Department of Immunology, University of North Carolina at Chapel Hill, Chapel Hill, North Carolina 27599, USA; 4 Department of Clinical Science, UT-Southwestern Medical Center, Dallas, Texas 75390, USA; 5 Department of Neuroscience, UT-Southwestern Medical Center, Dallas, Texas 75390, USA; 6 Eugene McDermott Center for Human Growth and Development, The University of Texas Southwestern Medical Center, Dallas, Texas 75390, USA

## Abstract

Tumours frequently activate genes whose expression is otherwise biased to the testis, collectively known as cancer–testis antigens (CTAs). The extent to which CTA expression represents epiphenomena or confers tumorigenic traits is unknown. In this study, to address this, we implemented a multidimensional functional genomics approach that incorporates 7 different phenotypic assays in 11 distinct disease settings. We identify 26 CTAs that are essential for tumor cell viability and/or are pathological drivers of HIF, WNT or TGFβ signalling. In particular, we discover that Foetal and Adult Testis Expressed 1 (FATE1) is a key survival factor in multiple oncogenic backgrounds. FATE1 prevents the accumulation of the stress-sensing BH3-only protein, BCL-2-Interacting Killer (BIK), thereby permitting viability in the presence of toxic stimuli. Furthermore, ZNF165 promotes TGFβ signalling by directly suppressing the expression of negative feedback regulatory pathways. This action is essential for the survival of triple negative breast cancer cells *in vitro* and *in vivo*. Thus, CTAs make significant direct contributions to tumour biology.

## Abstract (web summary)

Proteins usually expressed solely in the testes are often found over-expressed in cancer and are termed cancer testis antigens. Here, the authors use a comprehensive screening strategy to identify 26 cancer-testis antigens that promote tumorigenic behaviour.

## Graphical abstract

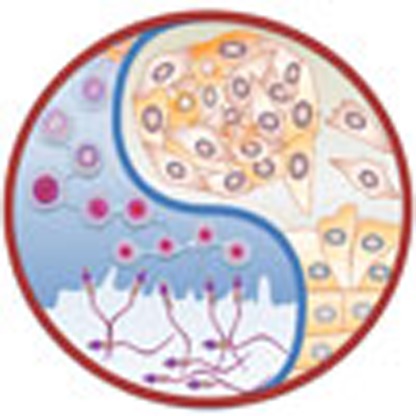

Cancer–Testis Antigens (CTAs) were originally discovered by employing patient-derived cytotoxic T-lymphocytes to identify the tumour antigens to which they were directed. Extensive analysis employing genetic, biochemical and differential gene expression profiling approaches has identified >250 genes classified as CTAs^1^. The testes are necessarily immune privileged as spermatogenesis generates auto-antigens long after the development of a competent immune system^2^. Thus, antigens derived from anomalously expressed testes proteins can evoke a cellular and/or humoral immune response and therefore these proteins are considered targets for immunotherapy^3^,^4^. In support of this, targeting the CTA, NY-ESO-1, using vaccination or adoptive T-cell transfer has exhibited efficacy in melanoma and synovial sarcoma patients, respectively^5^,^6^.

Given the extensive CTA compendium assembled and with immune targeting techniques gaining traction, attention is now turning to the function of CTAs in normal and diseased tissue. Twenty-four CTAs have been individually deleted in murine models. The majority of knockouts exhibit defects confined to gametogenesis. Specifically, CTAs can contribute to chromosome pairing during meiosis, sperm motility, translational regulation during sperm maturation, chromatin remodelling and transcriptional regulation^7^. With respect to tumorigenesis, a handful of CTAs have been implicated in centrosomal clustering, regulation of microtubule dynamics, p53 silencing and deflection of differentiation signalling^8^,^9^,^10^,^11^.

Based on these nascent indications that individual CTAs have tumorigenic activities, in this study we constructed an investigational pipeline to define the frequency and nature of CTA participation in tumorigenic behaviours. From within this cohort, we present two mechanistic examples that highlight the dramatically diverse functions CTAs can carry out in tumour cells. Foetal and Adult Testis Expressed 1 (FATE1) permits survival in the context of oncogenic stress by recruiting a previously uncharacterized E3 ligase, RNF183, to degrade the stress-sensing BH3-only protein, BCL-2-Interacting Killer (BIK). In comparison, ZNF165 drives the unrestrained activation of transforming growth factor β (TGFβ) signalling by directly inactivating the expression of negative feedback pathway regulators, SMURF2, SMAD7 and PMEPA1. These findings indicate extraordinary flexibility in tumour cell regulatory networks, rendering them vulnerable to corruption on aberrant expression of primordial gene-regulatory programmes.

## Results

### Functional analysis of CTAs

**Figure 1:**
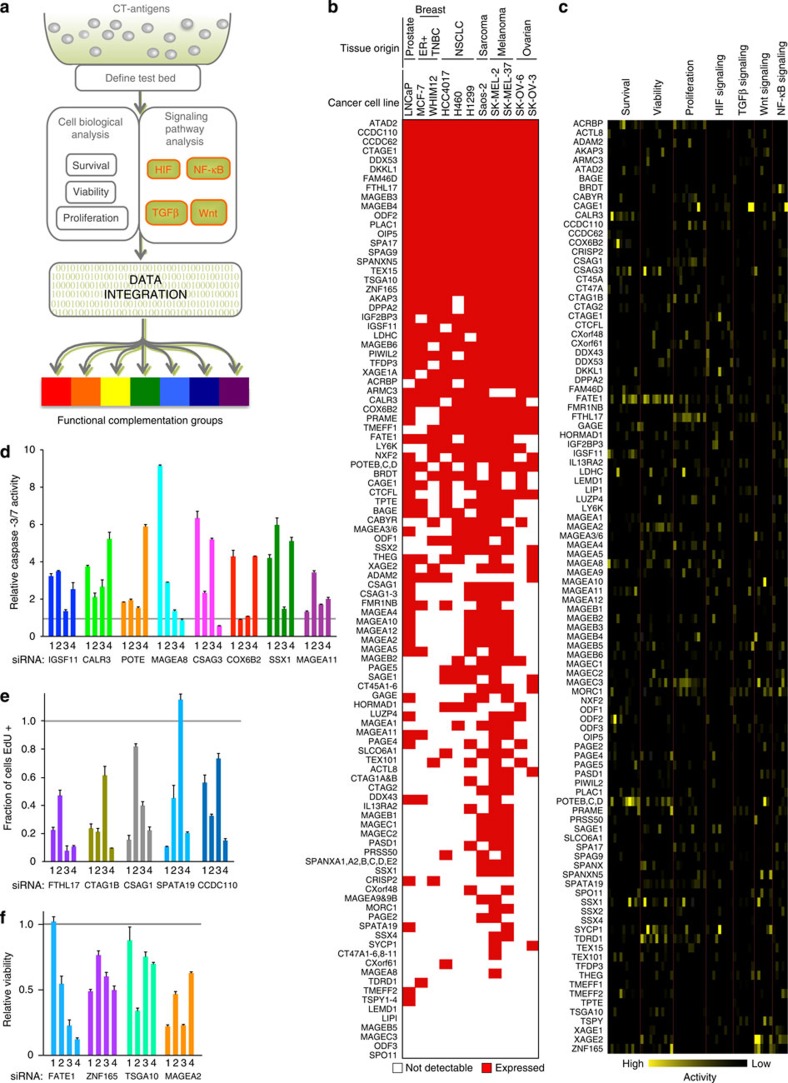
Platform for a multidimensional screen to interrogate CTA function. (**a**) Workflow for siRNA screen. (**b**) Presence (red) and absence (white) calls for all CTAs in indicated cell lines based on quantitative expression analysis. See Supplementary Data 2. (**c**) Z-scores for each screen were calculated and plotted for each CTA (left) for each assay and cell line (top, cell lines are hidden). (**d**–**f**) SiRNAs were transfected into cell lines, and 96 h post transfection APO (**d**), EdU (**e**) and CTG (**f**) assays were performed. Bars represent the average mean (*n*=2)±range. Grey line indicates activity of control siRNA. Numbers indicate independent siRNA sequences.

We designed an experimental platform to allow for broad-scale investigation of the mechanistic contribution of CTAs to tumour cell autonomous behaviours (Fig. 1a). One-hundred forty CTAs with documented expression in solid tumours were selected for this study (Supplementary Data 1). Given the notorious heterogeneity in expression of CTAs among tumours, we used quantitative expression profiling to identify 11 tumour-derived cell lines providing maximal representation of CTAs (135 CTAs) (Fig. 1b and Supplementary Data 2)^1^. These cell lines were derived from prostate, breast, ovarian, skin, non-small cell lung cancer (NSCLC) and bone tumours. Each cell line within this ‘testbed' exhibited a distinct pattern of CTA expression; however, most CTAs were present in >2 cell lines and 20% were expressed in all 11 cell lines (Fig. 1b).

To annotate tumorigenic CTAs, we individually depleted each CTA in each of the 11 cell lines and measured the consequences on viability, apoptosis and proliferation (Supplementary Fig. 1a–g). In addition, we measured the consequences of CTA depletion on the Hypoxia Inducible Factor (HIF), Wnt, TGFβ and Nuclear Factor Kappa-light-chain-enhancer of activated B cells (NF-κB) signalling pathways in a subset of cell lines using luciferase reporters (Supplementary Fig. 1b,h,i). These pathways were chosen because they are classic tumorigenic signalling cascades that are also essential during development and therefore, we reasoned were most likely to be affected by CTAs in tumour cells. Importantly, each luciferase signalling reporter exhibited a broad dynamic range on ligand-mediated stimulation in at least five testbed cell lines, providing an opportunity to examine CTA influence in multiple genetic backgrounds (Supplementary Fig. 1h,i). Raw data from each screen were normalized to internal non-targeting controls and a *z*-score was calculated for all CTAs in each assay and cell line (Fig. 1c and Supplementary Data 3).

### Multiple CTAs are essential for tumour cell viability

*S*hort interfering RNA (siRNA) pools targeting 17 CTAs that significantly affected apoptosis, viability or proliferation (defined as >1.5 s.d. from the mean of all CTAs) were retested using individual siRNAs. Fourteen pools contained two or more active siRNAs, suggesting on-target activity (Fig. 1d–f). Among these validated CTAs were members of the MAGE (A8 and A2), SSX (SSX1) and CTAG (CTAG1B) families, which have long been considered important vaccine targets^7^. Notably, CTAG1B (also known as NY-ESO-1), the CTA with clinical efficacy as an immunotherapeutic target, was required for tumour cell proliferation. We identified regulators of sperm oxidative phosphorylation, COX6B2, and sperm migration, CALR3, as essential for survival^12^,^13^. CTAs implicated in regulation of iron homeostasis, FTHL17, and mitochondrial adhesion in sperm, SPATA19, were required for proliferation^14^,^15^. Validated CTAs also included proteins with undefined contributions to spermatogenesis including IGSF11, CSAG1&3, CCDC110, ZNF165 and FATE1. Taken together, these results indicate that CTAs with diverse spermatogenic functions can be engaged and repurposed by the tumorigenic environment to support cell growth and division.

### FATE1 thwarts pro-apoptotic signalling

**Figure 2:**
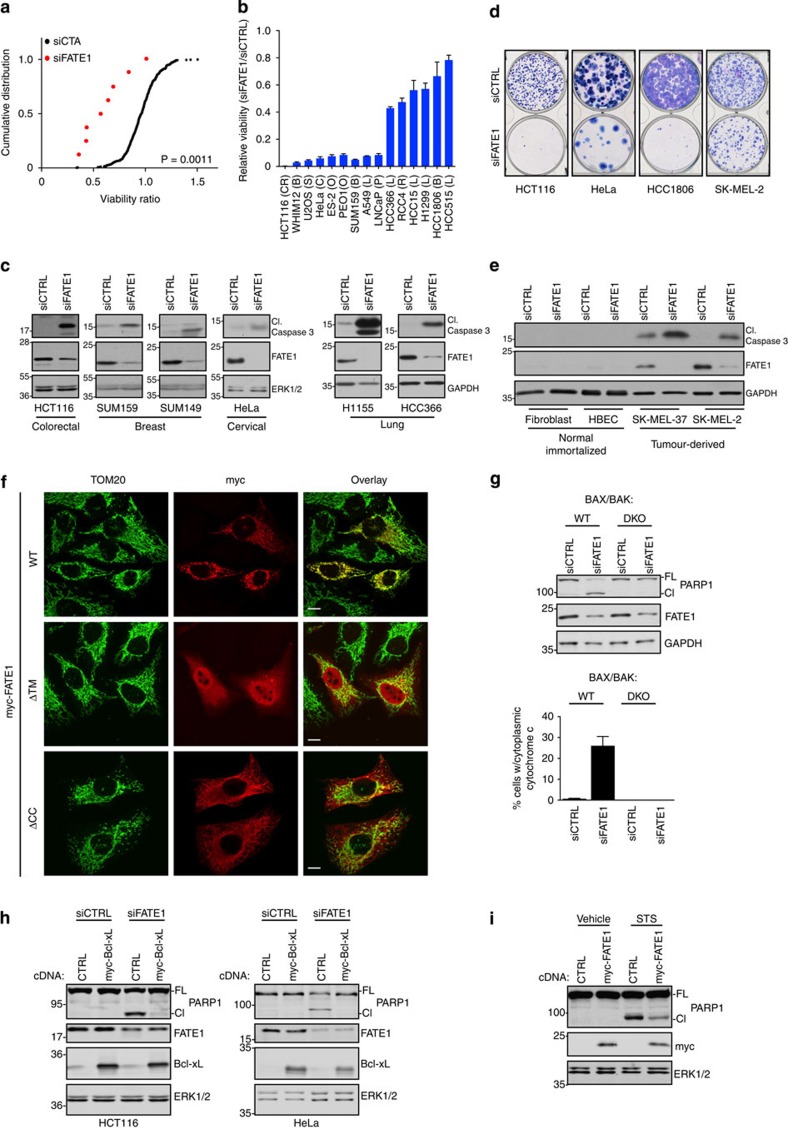
FATE1 supports tumour cell viability. (**a**) Distribution of siFATE1 viability ratios versus all other siCTAs in testbed cell lines with detectable FATE1 expression. Points represent mean of at least two independent assays. *P* value calculated by Kolmogorov–Smirnov test. (**b**) Viability assay in indicated cell lines 120 h post siRNA transfection. Bars represent mean viability relative to siCTRL (*n*=4)±s.d. B, breast; C, cervical; CR, colorectal; L, lung; S, osteosarcoma; O, ovarian; P, prostate; R, renal. (**c**) Whole cell lysates (WCLs) from indicated cell lines transfected with siCTRL or siFATE for 48 (HCT116), 72 (SUM159, SUM149, H1155) or 96 h (HeLa, HCC366) were immunoblotted with indicated antibodies. Data representative of a minimum of two independent assays. (**d**) Colony formation assays were performed 48 (HCT116) or 72 h after siRNA transfection. Data representative of two independent assays. (**e**) WCLs from indicated cell lines were immunoblotted as indicated 96 h after siRNA transfection. Data representative of two independent assays. (**f**) HeLa cells transfected with indicated cDNAs for 24 h were fixed, immunostained with indicated antibodies (top) and imaged with confocal microscopy. WT, wild-type; ΔTM, transmembrane deletion; ΔCC, coiled-coiled deletion. Scale bars, 10 μm. Data representative of two independent assays. TOM20 was used to visualize the mitochondria. (**g**) Top: 48 h after siRNA transfection, WCLs from indicated HCT116 cells (double knockout (DKO)) were immunoblotted with indicated antibodies. Data representative of two independent assays. Bottom: HCT116 cells were transfected and exposed to 10 μM Q-VD-OPh for 48 h, fixed and immunostained for cytochrome *c* and TOM20. Cytoplasmic cytochrome *c* was quantitated manually for ≥200 cells per experiment for each condition. Bars represent mean (*n*=3)±s.d. Cl, cleaved PARP1; FL, full-length PARP1. (**h**) WCLs from HCT116 or HeLa cells stably expressing a control construct (HCT116, empty vector; HeLa, pLPCX-GFP) or myc-Bcl-xL were immunoblotted with indicated antibodies 48 (HCT116) or 72 h (HeLa) post siRNA transfection. (**i**) H1299 cells stably expressing empty vector or myc-FATE1 were exposed to vehicle or 1 μM staurosporine for 6 h. WCLs were immunoblotted with indicated antibodies. Data representative of three independent assays.

The CTA that exhibited the most potent viability dependency in multiple genetic backgrounds was FATE1 (*P*=0.0011, Kolmogorov–Smirnov test) (Fig. 2a and Supplementary Data 3). Depletion of FATE1 led to a >30% reduction of viability in melanoma, breast, prostate and sarcoma settings (Supplementary Fig. 2a). Importantly, three of the four siRNAs in the original screening pool were sufficient to recapitulate the activity of the siRNA pool (Fig. 1f). We also returned FATE1 as essential for viability in a previous genome-wide loss-of-function screen in H1155 cells, a NSCLC cell line^9^. Given this broad penetrance, we further evaluated the tumour cell dependency on FATE1 by expanding our analysis to additional cell lines derived from colorectal, ovarian, sarcoma, breast, cervical and NSCLC cancers. While all cell lines were sensitive to FATE1 depletion, we identified a subset (HCT116, WHIM12, U2OS, HeLa, ES-2, PEO1, SUM159, A549, LNCaP), which exhibited an almost complete loss of viability 120 h post transfection with siFATE1 (Fig. 2b). In the cleaved caspase-3/7 survival screen, depletion of FATE1 elevated the activity of these executioner caspases greater than twofold, a phenotype that was also detectable by immunoblot in a range of tumorigenic settings and suggests that FATE1 is essential for tumour cell survival (Fig. 2c and Supplementary Fig. 2b). These observations corresponded with a potent loss in viability as assessed by colony formation assays in multiple tumorigenic backgrounds (Fig. 2d). Importantly, while FATE1 protein was detectable in all tumour-derived cell lines, it was low or undetectable in telomerase immortalized, non-tumorigenic fibroblasts and lung epithelial cells, where siFATE1 did not lead to cleavage of caspase-3 (Fig. 2e). Taken together, these data suggest that reactivation of FATE1 may be a survival dependency in the transformed environment irrespective of disease site.

Analysis of FATE1's secondary structure indicated a largely disordered protein with the exception of C-terminal coiled-coil (CC) and transmembrane domains (TM), which exhibit 29% identity and 55% similarity to mitochondrial fission factor (Supplementary Fig. 2c–e)^16^. Consistent with this similarity, myc-FATE1 localized to the mitochondria with limited presence in other organelles (Fig. 2f and Supplementary Fig. 2f–h). Mitochondrial localization was dependent on both the transmembrane and CC domains, as their deletion resulted in nuclear and endoplasmic reticular localization, respectively (Fig. 2f and Supplementary Fig. 2g). Given that mitochondria are the central hub of apoptotic signalling, we further analysed the role of key apoptotic regulators in siFATE1-induced death. Depletion of FATE1 in HCT116 cells induced cleavage of PARP1 and cytochrome *c* release, a phenotype that was absent in BAX/BAK null HCT116 cells (Fig. 2g). Overexpression of the anti-apoptotic Bcl-2 family member, Bcl-xL, also rescued cell death following siFATE1 (Fig. 2h). Consistent with a general role in deflecting apoptosis, we found that cells overexpressing FATE1 exhibited attenuated PARP1 cleavage following staurosporine challenge (Fig. 2i).

**Figure 3:**
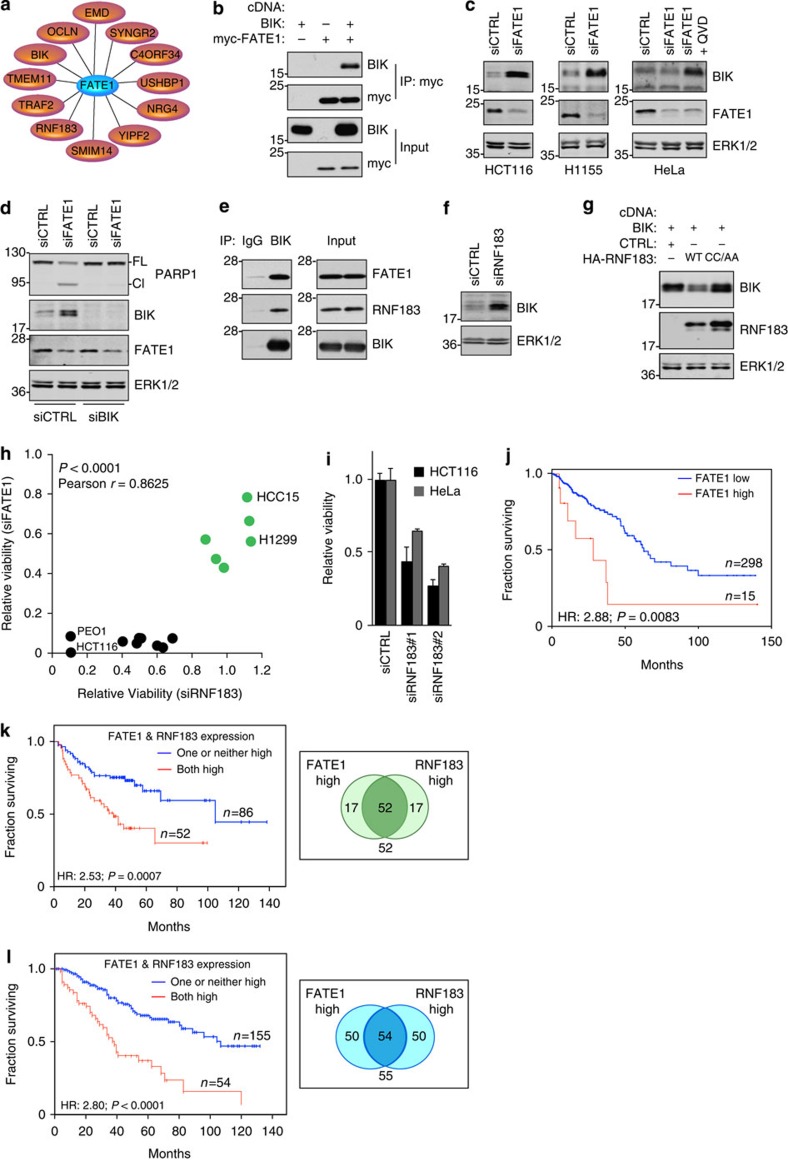
FATE1 and the E3 ligase RNF183 impact the stability of the apoptotic effector BIK. (**a**) Interaction data for FATE1 based on yeast two-hybrid proteomics analyses. (**b**) Sixteen hours after transfection in HEK293T cells, lysates (pH 8.0) were incubated with myc antibodies, washed in NDLB and immunoblotted with indicated antibodies. Data representative of three independent assays. (**c**) WCLs were collected 48 (HCT116) or 72 h (H1155 and HeLa) following siRNA transfection and immunoblotted as indicated. +QVD, cells were exposed to 10 μM of the pan-caspase inhibitor Q-VD-OPh for the duration of the experiment. Data representative of at least two independent assays. (**d**) HCT116 cells were transfected for 24 h with siCTRL or siBIK then transfected with siCTRL or siFATE for an additional 48 h. WCLs were collected and immunoblotted as indicated. Data representative of three independent assays. (**e**) Twenty hours post transfection with BIK-L61G, FATE and HA-RNF183 cDNAs, HEK293T cells were lysed in NDLB, immunoprecipitated and immunoblotted as indicated. Data representative of two independent assays. (**f**) WCLs from HCT116 cells transfected with indicated siRNAs for 72 h were immunoblotted with indicated antibodies. Data representative of two independent assays. (**g**) WCLs from H1299 cells transfected with indicated cDNAs for 24 h were immunoblotted as indicated. WT, wild-type. CC/AA, C13A/C59A. Data representative of three independent assays. (**h**) Cell lines from Fig. 2b were transfected for 120 h with siFATE (*y*-axis) or siRNF183 (*x*-axis) and cell viability was measured via CTG. Dots represent mean of four independent assays. (**i**) Viability assays was performed 96 h after siRNA transfection in indicated cell lines. ‘#' indicates independent siRNA pools. Bars represent mean (*n*=3)±s.d. (**j**) Kaplan–Meier (KM) survival curves from TCGA colorectal adenocarcinoma patients. HR and *P* value calculated by Cox Regression Analysis. (**k**) KM survival curves of patients from GSE42127 as a function of high FATE1- and RNF183-expressing tumours. Venn diagram represents proportion of patients in each group. HR and *P* value calculated by Cox Regression Analysis. (**l**) as in **k** with patients from GSE8894.

To further elaborate the mechanisms of FATE1's anti-apoptotic function, we examined FATE1-interacting partners previously reported by large-scale proteomics studies (Fig. 3a)^17^,^18^. Among the dozen interactors, was the founding member of the pro-apoptotic BH3-only family, BIK^19^, which we found associates with FATE1 in mammalian cells (Fig. 3b). BIK is reported to promote cell death by mobilizing cytochrome *c*, which is released on a parallel signal from NOXA^20^. BIK can be induced by genotoxic stress, E2F stimulation, cytokines (IFNγ and TGFβ), IgM ligation and oestrogen starvation^21^. BIK expression is frequently upregulated in tumours as compared with normal tissues (Supplementary Fig. 3a); however, BIK protein is low to undetectable in tumours due to proteasome-mediated turnover, indicating that tumour cells must adapt mechanisms to prevent BIK protein accumulation for tumour cell survival^21^,^22^,^23^. On depletion of FATE1, we observed a strong stabilization of BIK protein in multiple tumorigenic settings (Fig. 3c). Significantly, depletion of BIK rescued siFATE-induced apoptotic signalling (Fig. 3d), which led us to hypothesize that FATE1 may be involved in the regulation of BIK stability. While FATE1 does not have any identifiable E3 ligase domains, we found that it associated with an uncharacterized E3 ligase, RNF183, in proteomics analysis and in intact cells (Fig. 3a and Supplementary Fig. 3b)^18^. RNF183 is an ER-localized protein that contains a C3HC4−RING domain, which was required for autoubiquitination activity (Supplementary Fig. 3c–e). In addition, we found that BIK associated with both FATE1 and RNF183 in intact cells, suggesting a functional relationship between these proteins (Fig. 3e). Indeed, RNF183 depletion increased BIK protein accumulation (Fig. 3f and Supplementary Fig. 3f). Conversely, overexpression of wild-type RNF183, but not the C13A/C59A ligase-dead mutant, reduced expression of exogenous BIK, indicating that RNF183's E3 activity negatively regulates BIK protein accumulation (Fig. 3g and Supplementary Fig. 3e). Given its potent effect on the stabilization of BIK, we evaluated the consequences of RNF183 depletion on cell viability. We observed a striking correlation between siFATE1 and siRNF183 sensitivity in a panel of tumour-derived cell lines (Fig. 3h,i). These findings indicate that FATE1 and RNF183 collaborate to restrain BIK protein levels, and thus escape from apoptotic signalling.

To examine the consequences of FATE1 expression in patient tumours, we examined the Cancer Genome Atlas (TCGA) colorectal data set, given that depletion of FATE1 was most potent in the HCT116 colorectal cell line. Here we found that elevated levels of FATE1 were associated with significantly poorer survival (hazard ratio (HR)=2.88; *P*=0.0083; Cox Regression) (Fig. 3j). In NSCLC, FATE1 expression alone did not portend poor survival; however, we observed that patients with tumours expressing elevated FATE1 and RNF183 were at higher risk for poor survival (HR=2.53; *P*=0.0007; Cox Regression) as compared with those patients with one or neither expressed (Fig. 3k). In a separate NSCLC data set, the majority of tumours with high FATE1 expression also exhibited elevated RNF183 expression, and again, high expression of both genes predicted shortened overall survival time (HR=2.80; *P*<0.0001; Cox Regression) (Fig. 3l). The frequency of co-expression of RNF183 and FATE1 along with their correlation with poor outcome reinforces the notion that these proteins are functioning in human tumours to promote survival.

### CTAs are novel regulators of tumorigenic signalling cascades

**Figure 4:**
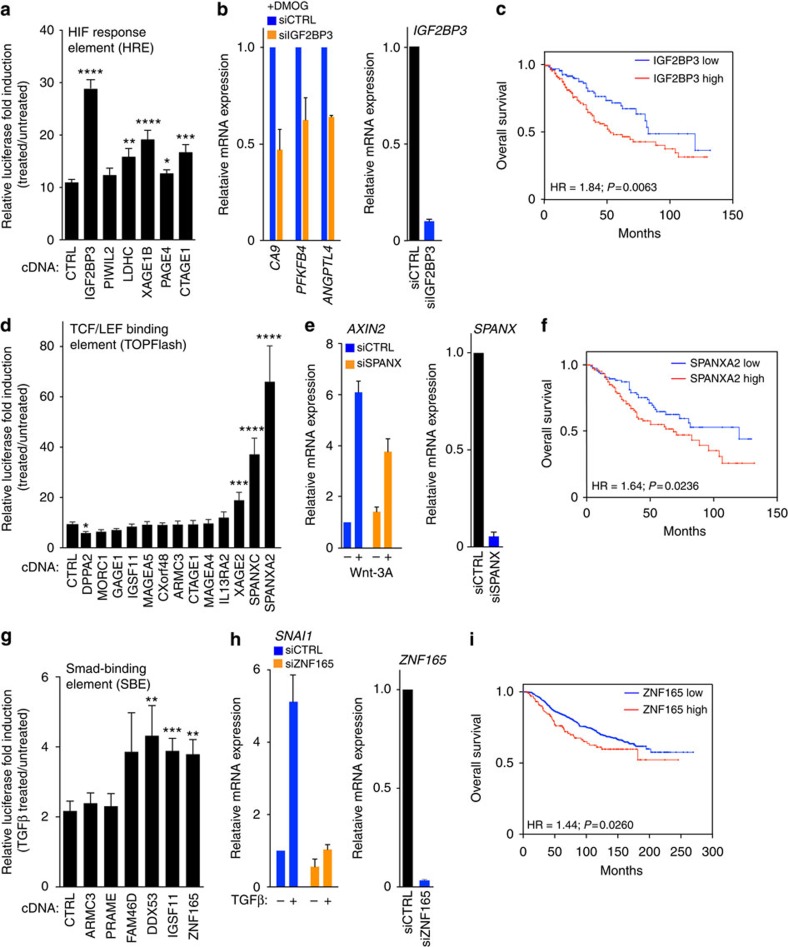
CTA expression is sufficient to activate oncogenic signalling cascades. (**a**) Indicated cDNAs were co-transfected with the HRE luciferase reporter into HEK293T cells. Following 10 h of stimulation with DMOG, luciferase activity was measured. Bars represent mean (*n*≥16)±s.e.m. *P* values calculated by unpaired Student's *t*-test. ****P≤0.0001, ***P≤0.0005, **P≤0.01, *P<0.05. (**b**) H1299 cells were transfected with indicated siRNAs for 48 h prior to stimulation for 16 h with 1 mM DMOG. Quantitative PCR (qPCR) was used to quantitate relative mRNA expression of indicated genes normalized to DMOG-stimulated siCTRL. Bars represent mean (*n*=2)±range. (**c**) KM survival curves of patients from GSE42127 as a function of IGF2BP3 high- and low-expressing tumours. HR and *P* value calculated by Cox Regression Analysis. (**d**) Indicated cDNAs were co-transfected with the Wnt luciferase reporter into HEK293T cells. Following 10 h of 500 ng ml^−1^ Wnt-3a stimulation, luciferase activity was measured. Bars represent mean (*n*≥8)±s.e.m. *P* values calculated by unpaired Student's *t*-test as in **a**. (**e**) Saos-2 cells were transfected with indicated siRNAs for 72 h and exposed to 500 ng ml^−1^ Wnt-3a for 3 h. QPCR was then performed to quantitate AXIN2 and SPANX mRNA expression. Bars represent mean (*n*=2)±range. (**f**) As in **c** except the KM plots are a function of SPANXA2 expression levels. (**g**) Indicated cDNAs were co-transfected with the SBE luciferase reporter in HEK293T cells. Following 24 h of 100 ng ml^−1^ TGFβ stimulation, luciferase activity was measured. Bars represent mean (*n*≥4)±s.e.m. *P* values calculated by unpaired Student's *t*-test as in **a**. (**h**) WHIM12 cells were transfected with indicated siRNAs for 48 h and then exposed to 100 ng ml^−1^ TGFβ for 3 h. QPCR was then performed to quantitate SNAI1 and ZNF165 mRNA expression. Bars represent mean (*n*=2)±range. (**i**) As in **c**, except KM plots are using patients from the METABRIC data set and are a function of ZNF165 expression.

We next turned our investigation to the CTAs returned from screens of regulatory signalling modules. We prioritized siRNAs from these screens based on the extent and genetic penetrance of their activity, which returned a large set (>10) of CTAs as putative regulators of each pathway (See Methods and Supplementary Data 4). We reasoned that this large set could be due to multiple indirect mechanisms impinging on these signalling modules. Therefore, we asked whether CTAs returned from these screens were sufficient to activate the pathways for which they were required. Of the six CTAs tested from the HIF screen, LDHC, XAGE1B, PAGE4, CTAGE1 and IGF2BP3 were all sufficient to enhance dimethyloxalylglycine-*N*-(methoxyoxoacetyl)-glycine methyl ester (DMOG)-mediated activation of the HIF response element (HRE) reporter (Fig. 4a). We further examined IGF2BP3, which exhibited the strongest effect. IGF2BP3 is expressed in trophoblasts during the first trimester of pregnancy, and low expression of IGF2BP3 is associated with attenuated trophoblast invasion and preeclampsia^24^. Measurement of canonical HIF targets showed that IGF2BP3 depletion attenuated the endogenous expression of HIF target genes in H1299 lung adenocarcinoma cells (Fig. 4b). Our findings here suggest that reactivation of IGF2BP3 could inappropriately activate the HIF pathway, promoting invasive and metastatic behaviour and reducing overall survival. Indeed, in NSCLC, elevated IGF2BP3 portends poor overall survival (HR=1.84; *P*=0.0063; Cox Regression) (Fig. 4c).

Of the 13 CTAs retested from the Wnt screen, SPANXC and SPANXA2 strongly promoted Wnt-3a-induced TCF4 response element reporter activity (Fig. 4d). Depletion of multiple SPANX family members, which was necessary due to high sequence similarity, inhibited expression of the canonical Wnt target, AXIN2 (Fig. 4e). In NSCLC, we found that high SPANXA2 expression correlated with poor overall survival (HR=1.64; *P*=0.0236; Cox Regression) (Fig. 4f).

We retested six CTAs returned from the NF-κB screen; however, we were unable to detect a gain-of-function phenotype, suggesting that these CTAs may support processes that indirectly impinge on NF-κB signalling.

Of six CTAs tested from the TGFβ screen, DDX53, IGSF11 and ZNF165 were sufficient to promote TGFβ-induced SMAD binding element (SBE) reporter activity (Fig. 4g). We analysed ZNF165 in more detail because its depletion was also selectively lethal in the TGFβ-dependent, triple negative breast cancer (TNBC) cell line, WHIM12 (Fig. 1c and Supplementary Fig. 4a,b). Depletion of ZNF165 inhibited activation of the canonical TGFβ target gene, SNAI1, in WHIM12 cells (Fig. 4h). Moreover, expression of ZNF165 was correlated with poor survival in breast cancer patients (HR=1.44; *P*=0.0260; Cox Regression) (Fig. 4i).

### ZNF165 attenuates negative feedback of the TGFβ pathway

**Figure 5:**
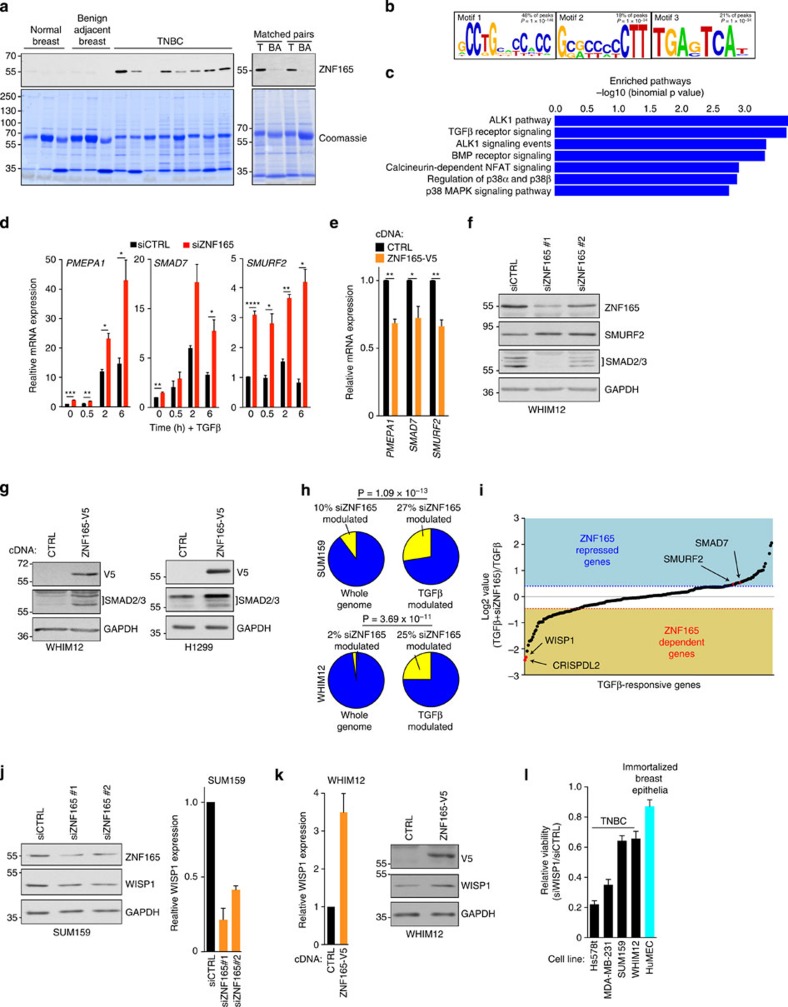
ZNF165 promotes TGFβ pathway activation and drives expression of the WISP1 oncogene. (**a**) Immunoblots and Coomassie stain of TNBC tissues. BA, benign-adjacent; N, normal; T, tumour. (**b**) ZNF165-V5 binding motifs. (**c**) Pathways enriched for WHIM12-ZNF165-V5-associated genes. *P* value calculated using a binomial test. (**d**) WHIIM12 cells were transfected for 48 h with indicated siRNAs, stimulated with vehicle or 10 ng ml^−1^ of TGFβ for indicated times and mRNA expression quantitated by qPCR. Values are graphed relative to siCTRL at 0 min. Bars represent mean (*n*=3)±s.e.m. *P* values calculated by one-tailed, unpaired Student's *t*-test for zero time points and two-tailed, paired Student's *t*-test for all others. ****P≤0.0001, ***P≤0.0005, **P≤0.01, *P<0.05. (**e**) Relative mRNA expression of indicated genes was quantitated by qPCR in WHIM12-HcRed (CTRL) or WHIM12-ZNF165-V5 cells. Bars represent mean (*n*=3)±s.e.m. *P* value calculated from one-tailed Student's *t*-test. *P≤0.05, **P≤0.01. (**f**) Immunoblots of WHIM12 WCLs cells transfected with indicated siRNAs for 48 h. Data representative of three independent assays. (**g**) Left: WHIM12-HcRED (CTRL) and WHIM12-ZNF165-V5 WCLs were immunoblotted as indicated. Right: 48 h after transient cDNA transfection, H1299 WCLs were immunoblotted as indicated. Data representative of three independent assays. (**h**) Fraction of genes modulated following siZNF165 based on significant analysis of microarrays analysis (criteria: false discovery rate≤10%, Student's *t*-test *P*≤0.05). *P* values calculated by hypergeometric distribution analysis. (**i**) TGFβ-induced genes rank ordered based on fold change following siZNF165 and exposure to 10 ng ml^−1^ TGFβ.(**j**) Left: WCLs from SUM159s transfected with indicated siRNAs for 48 h were immunoblotted with indicated antibodies. Data representative of two independent assays. Right: mRNA was collected in parallel and WISP1 expression quantitated by qPCR. Bars represent mean (*n*=3)±range. (**k**) Left: RNA was isolated from WHIM12 cells stably expressing ZNF165-V5 or HcRED (CTRL) and relative WISP1 mRNA was quantitated by qPCR. Bars represent mean (*n*=2)±range. Right: WCLs were immunoblotted with indicated antibodies. Data representative of three independent assays. (**l**) Viability assay following siWISP1. Values are graphed relative to siCTRL. Bars represent mean (*n*=3)±s.d.

Based on ZNF165's correlation with poor outcome in breast cancer patients, its requirement for viability of WHIM12s and its augmentation of TGFβ pathway activity, we chose to further investigate how ZNF165 engages TGFβ signalling in the setting of TNBC. As ZNF165 mRNA expression is elevated in multiple tumour types, we first evaluated ZNF165 protein expression in 10 TNBC, 3 normal and 5 benign-adjacent tissues, including 2 tumour and benign matched pairs (Fig. 5a. and Supplementary Fig. 4c)^25^. ZNF165 protein expression was detectable in 9 of the 10 tumour tissues tested, with minimal accumulation in benign or normal breast-derived samples (Fig. 5a). ZNF165 is an uncharacterized member of the SCAN-(C_2_H_2_)_*n*_ sub-family of zinc finger proteins and contains a SCAN heterodimerization domain and five C_2_H_2_ motifs, which are canonical zinc finger domains that mediate association with DNA (Supplementary Fig. 4d)^26^,^27^. ZNF165 also localizes to the nucleus in tumour cells and associates with nine proteins with gene-regulatory activity (Supplementary Fig. 4e,f)^18^,^28^. Given these indications of transcriptional regulatory activity, we performed chromatin immunoprecipitation followed by next-generation sequencing (ChIP-Seq) analysis in WHIM12 cells stably expressing ZNF165-V5 to identify putative target genes. This analysis returned 281 ZNF165 binding sites associated with 447 genes (Supplementary Data 5). *De novo* motif enrichment identified three motifs that comprised ∼90% of these binding sites (Fig. 5b). Genomic Regions Enrichment of Annotation Tool (GREAT) analysis revealed that genes associated with ZNF165 peaks are significantly enriched in the TGFβ signalling pathway (23 genes; *q*=0.00696, binomial test) (Fig. 5c, Supplementary Data 5). This gene set contained multiple negative regulators of TGFβ signalling including SMURF2, SMAD7 and PMEPA1, which we validated as true positives by ChIP–quantitative PCR (qPCR) (Supplementary Fig. 4g)^29^,^30^,^31^,^32^,^33^. As ZNF165 was sufficient to activate the TGFβ reporter, we examined the consequences of ZNF165 on expression of these negative regulators of TGFβ signalling. First, we verified that SMAD2/3 was phosphorylated in response to TGFβ stimulation in WHIM12 cells (Supplementary Fig. 4h). As shown in Fig. 5d, TGFβ stimulation increased accumulation of PMEPA1, SMAD7 and SMURF2 mRNA, which was enhanced on depletion of ZNF165 (Fig. 5d). Conversely, we found that WHIM12 cells with stable overexpression of ZNF165-V5 displayed attenuated PMEPA1, SMAD7 and SMURF2 mRNA expression, suggesting that ZNF165 inhibits expression of these genes directly (Fig. 5e). An increase in the E3 ligase, SMURF2, would be predicted to lead to a loss of SMAD2/3 protein, a major signal transducer of TGFβ signalling^29^,^33^. Indeed, we found that depletion of ZNF165 with two independent siRNA pools in WHIM12 cells led to enhanced SMURF2 accumulation and attenuation of SMAD2/3 protein (Fig. 5f). We also observed this phenotype, as well as a decrease in phosphorylation of SMAD2/3, in a second TNBC- and TGFβ-dependent cell line, SUM159 (Supplementary Fig. 4b,i). Conversely, WHIM12-ZNF165-V5 cells exhibited an accumulation of SMAD2/3 protein as compared with control infected WHIM12s (Fig. 5g). Transient overexpression of ZNF165 in H1299 cells (a NSCLC-derived cell line chosen for its high level of transient transfection efficiency not attainable in the TNBC setting) was also sufficient to enhance SMAD2/3 protein accumulation (Fig. 5g). To further evaluate the consequences of ZNF165 on the global TGFβ regulatory network, we performed whole genome expression profiling in SUM159 and WHIM12 cells depleted of ZNF165 and stimulated with TGFβ (Supplementary Data 6). In both settings, ∼25% of all TGFβ modulated genes were affected by ZNF165 depletion, representing an enrichment with a probability of ≥3.69 × 10^−11^ by random chance according to hypergeometric distribution analysis (Fig. 5h). In agreement with the ChIP analysis, canonical TGFβ targets that are negative feedback regulators (SMURF2 and SMAD7) were activated on ZNF165 depletion (Fig. 5i)^29^,^32^,^33^. ZNF165 was required for the expression of 30 TGFβ-induced genes, many of which mediate neoplastic processes (GPR124, FGD4, WISP1, DPYSL3, USP2, CRYAB and RASGRP1)^34^,^35^,^36^,^37^,^38^,^39^,^40^,^41^. One of the most dramatically repressed genes in both WHIM12 and SUM159 cells was WISP1, a poorly characterized oncogene that promotes growth and survival in colon cancer (Fig. 5i and Supplementary Data 6)^36^,^42^,^43^. We find that ZNF165 depletion leads to a reduction in WISP1 protein accumulation (Fig. 5j). Overexpression of ZNF165 is sufficient to enhance WISP1 mRNA and protein accumulation and stimulate the activity of luciferase fused to the WISP1 promoter (Fig. 5k and Supplementary Fig. 4j). Furthermore, WISP1 is essential for viability in multiple TNBC settings (Fig. 5l and Supplementary Fig. 4k). Taken together, this analysis indicates that ZNF165 expression directly promotes TGFβ pathway activity by repressing negative feedback and leads to the expression of target oncogenes essential for TNBC.

### ZNF165 is essential for TNBC survival *in vitro* and *in vivo*

**Figure 6:**
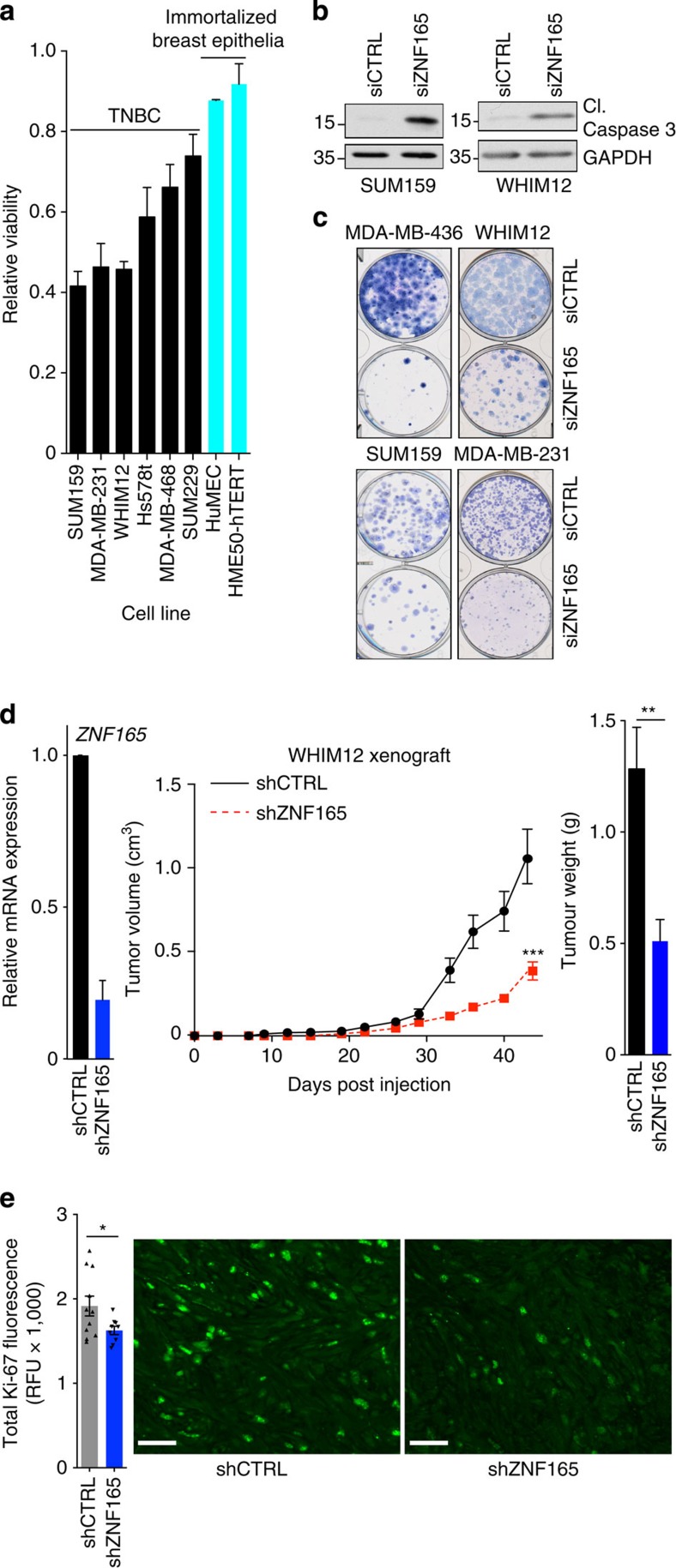
ZNF165 supports TNBC tumour cell viability. (**a**) Viability assays in indicated cells lines following siZNF165. Values represent viability relative to siCTRL. Bars represent mean (*n*=3)±s.d. (**b**) SUM159 and WHIM12 cells were transfected with indicated siRNAs for 72 h and 48 h, respectively, and WCL's were immunoblotted as indicated. Data representative of two independent assays. (**c**) Colony formation assays performed in TNBC cell lines 48 h after transfection with siZNF165. Data representative of at least two independent assays. Cell lines are indicated across the top. (**d**) Left: RNA from WHIM12 cells expressing shCTRL or shZNF165 was isolated and qPCR was used to quantitate relative ZNF165 mRNA. Bars are mean (*n*=2)±range. Middle: growth curves of cells from left panel injected s.c. and measured at indicated time points. Points represent average volume of shCTRL (*n*=11) or shZNF165 (*n*=9)±s.e.m., *P* value calculated with unpaired, two-tailed Student's *t*-test. ***P≤0.005. Right: At the study endpoint (middle panel), tumours were excised and weighed. Bars represent mean of tumour weights±s.e.m., *P* value calculated with unpaired, two-tailed Student's *t*-test. **P≤0.01. (**e**) Left: Tumours from **d**, (middle panel) were stained for Ki-67. Bars represent mean of total fluorescence across three randomly chosen fields for each tumour±s.e.m. *P* value calculated with unpaired, two-tailed, Student's *t*-test. *P≤0.05. Right: Representative images from Ki-67 (green) stained sections. Scale bars, 50 μm.

We next elaborated on our initial discovery that ZNF165 was essential for the viability of WHIM12 TNBC cells by assessing this phenotype in a larger cohort of tumour and normal, immortalized breast epithelial cells. Here we found that depletion of ZNF165 selectively reduced viability in a subset of TNBC cell lines (Fig. 6a and Supplementary Fig. 4l). Depletion of ZNF165 also led to cleavage of caspase-3 and a decrease in long-term growth as observed by colony formation assays (Fig. 6b,c). To next examine the role of ZNF165 during tumorigenesis, we assessed the consequences of ZNF165 depletion in a mouse xenograft model. WHIM12 cells stably infected with shCTRL or shZNF165 were injected into immunocompromised mice and tumour growth monitored for 7 weeks. In comparison to the control shRNA group, tumours from shZNF165 mice were attenuated in growth and exhibited reduced Ki-67 staining (Fig. 6d,e). Taken together, these findings suggest that ZNF165 promotes tumorigenesis by activating the TGFβ pathway, which in TNBC, is essential for tumour cell survival potentially in part through activation of the WISP1 oncogene.

## Discussion

Intriguing correlative associations between gametogenesis and tumorigenesis have been noted for over 100 years. For example, tumour cells can produce trophoblastic hormones at sufficient levels to be used as a serum marker for tumour detection and recurrence^44^. In addition, gene products whose expression is otherwise restricted to reproductive tissues are frequently re-expressed in a range of tumour types. However, despite widespread activation in tumours, a global investigation into the contribution of these proteins to neoplastic behaviours has been lacking. Here by integrating findings from a multi-faceted, comprehensive platform we find that CTAs engage divergent mechanisms in the tumorigenic regulatory network to promote cancer.

We uncovered multiple CTAs that are essential for tumour cell viability. These CTAs have a diversity of known functions within sperm and therefore likely buttress different aspects of the tumorigenic platform. In particular, we define FATE1 in promoting tolerance to cellular stress, which would otherwise lead to cellular demise. Successful tumorigenesis requires the circumvention of otherwise fatal fail-safe mechanisms and/or an adaption to unfavourable physiological conditions. Prototypical examples include thwarting oncogene-induced pro-apoptotic signalling, DNA damage stress, toleration of proteotoxic stress due to aneuploidy and buffering of oxidative stress resulting from altered mitochondrial function^45^,^46^,^47^. FATE1 may represent one mechanism by which tumour cells overcome these barriers by promoting the degradation of second messengers in this signalling system. Broadly these findings suggest that the engagement of lineage-specific gene products may be a generalizable mechanism to overcome barriers to transformation.

Our studies indicate that CTAs can be sufficient to activate oncogenic signalling modules. The capacity of ZNF165 to repress negative feedback of TGFβ signalling highlights how the expression of gametogenic genes in a somatic cell can wreak havoc on normal homeostatic regulatory mechanisms. Importantly, ZNF165 appears to be regulating the expression of a number of proteins, which are implicated in tumour cell survival. Further studies to determine the nature of ZNF165-mediated gene regulation *in vivo* will be necessary to better understand how ZNF165 promotes tumour progression. Importantly, our study of ZNF165 reveals a critical contribution of an understudied oncogene, WISP1, to TNBC, highlighting how elaboration of CTA function can reveal cryptic aspects of the tumour cell regulatory environment. The pro-tumorigenic features of TGFβ in late stage disease have made it a high value intervention target, particularly in TNBC; however, TGFβ is a pleiotropic cytokine with important roles in normal physiology, thereby limiting the efficacy of direct inhibition^48^. Our results suggest that ZNF165 may represent a mechanism to inhibit TGFβ signalling in a tumour cell-specific manner. This finding is also potentially generalizable to additional signalling pathways as we find that the CTAs, IGF2BP3 and SPANXA2 are sufficient to promote ligand-stimulated activation of HIF and Wnt signalling, respectively.

In summary, our findings provide a comprehensive understanding of the phenotypes conferred by CTAs when aberrantly expressed in the tumorigenic regulatory environment. The implications of these findings are twofold. First, the ectopic expression of CTAs in a somatic cell can impart a neomorphic function that may confer a selective advantage during tumorigenesis. Thus, annotation of CTA function reveals new aspects of tumour biology not previously appreciated, which could answer long-standing questions as to how tumour cells acquire specific features (for example, suppressing death signalling, activating epithelial-to-mesenchymal transition). Second, CTAs have long been considered ideal targets for anti-cancer vaccines or adoptive T-cell transfer. However, no objective criteria has been established for selecting CTAs to therapeutically target. We propose that CTAs with tumorigenic functions are the best candidates as they are most likely expressed in the majority of tumour cells and loss of expression would be detrimental to cancer cell survival.

## Methods

### Cell lines

Cell lines were obtained from American Tissue Type Collection (ATCC) or John Minna (UT-Southwestern (UTSW)) except for: SK-MEL-2 (the National Cancer Institute); SK-MEL-37 and SK-OV-6 (Lloyd Old, Ludwig Institute); SUM159, SUM229 and SUM149 (Asterand); HuMEC (Charles Perou, the University of North Carolina at Chapel Hill, (UNC)); HME50-hTERT, Fibroblasts (BJ) (Jerry Shay, UTSW); WHIM12 (Matthew Ellis, Baylor College of Medicine); ES-2 (Rolf Brekken, UTSW); PEO1 and U2OS (Michael White, UTSW); HEK293GP, HCC1806, Hs578t, MDA-MB-468 and MDA-MB-231 (Grey Pearson, UTSW); RCC4 (William Kim, UNC); HCT116 (Cyrus Vaziri, UNC), and HCT116-BAX^−/−^BAK^−/−^ DKO (Bert Vogelstein, Johns Hopkins University). All cell lines were cultured in provider's recommended medium. Because SK-OV-6 is on the ICLAC list of misidentified cell lines, short tandem repeat profiling was used to validate the line used in this study^49^. SK-OV-6 was used because it has previously been demonstrated to express a number of CTAs^1^. Cells were evaluated for mycoplasma contamination by DAPI stain for extra-nuclear DNA.

### Antibodies and reagents

Antibodies used for immunoblotting were as follows: Santa Cruz Biotechnology: GAPDH (sc-51907; 1:1,000), HA (sc-805; 1:500 and sc-7392; 1:500), TOM20 (sc-11415; 1:500), BIK (sc-10770; 1:500 and sc-1710; 1:250), c-Myc (sc-40; 1:1,000 and sc-789; 1:500), ERK1/2 (sc-93; 1:1,000), Ubiquitin (sc-8017; 1:250), and FATE1 (sc-101220; 1:1,000); Sigma: FATE1 (HPA034604; 1:2,000) and RNF183 (SAB2106627; 1:1,000); Cell Signaling Technology: Cleaved Caspase-3 (9661; 1:500), PARP1 (9532; 1:1,000), Bcl-xL (2764; 1:5,000) and Phospho-SMAD2 (pSMAD2/3) (Ser465/467)/SMAD3 (Ser423/425) (8828; 1:1,000); Abcam: WISP1 (ab10737; 1:1,000), FATE1 (ab111486; 1:1,000) and SMURF2 (ab53316; 1:1,000); ZNF165 (H00007718; 1:1,000; Novus Biologicals); SMAD2/3 (07–408; 1:1,000; Millipore); V5 (R960; 1:1,000; Life Technologies). Antibodies for immunofluorescence were as follows: c-Myc (sc-40; 1:100; Santa Cruz Biotechnology), TOM20 (sc-11415; 1:500; Santa Cruz Biotechnology), GM130 (ab31561; 1:100; Abcam), cytochrome *c* (556432; 1:200; BD Biosciences), HA (MMS-101R; 1:100; Covance), Catalase (219010; 1:1,000; Calbiochem), V5 (R960; 1:300), Calnexin (ADI-SPA-860; 1:100; Enzo), COX IV (4850; 1:125; Cell Signaling Technology), and β-tubulin (T5293; 1:100; Sigma).

Chemicals and reagents were purchased from the following manufacturers: DMOG (Caymen Chemical); Wnt-3a (R&D Systems); tumour necrosis factor-α (TNFα) (Promega); transforming growth factor-β (TGFβ) (Cell Signaling); pan-caspase inhibitor, (3S)-5-(2,6-difluorophenoxy)-3-[[(2S)-3-methyl-2-(quinoline-2-carbonylamino)butanoyl]amino]-4-oxopentanoic acid (Q-VD-OPh) (Sigma). siRNAs were obtained from GE Healthcare (siGENOME siRNA) or Sigma (Mission siRNA). Control (CTRL) siRNAs were either non-targeting control (GE Healthcare) or targeted genes DLNB14, FNDC3B or APOL6. CellTiter-Glo (CTG), Apo-ONE Homogenous Caspase-3/7 (APO), ONE-Glo Luciferase Assay System and Dual Glo Luciferase Assay System were purchased from Promega. Lipofectamine RNAiMAX and the Click-iT EdU (5-ethynyl-2′-deoxyuridine) Alexa Fluor 488 Imaging kit were purchased from Thermo Fisher Technologies. DharmaFECT reagents and DharmaFECT Duo were purchased from GE Healthcare Life Sciences. TRIzol, Opti-MEM and Hoechst 3342, trihydrochloride, trihydrate and MitoTracker Red CMXRos (C32H32Cl2N2O) were obtained from Thermo Fisher Scientific.

### Expression plasmids and mutagenesis

Unless otherwise specified full-length complementary DNAs (cDNAs) were obtained from the CCSB-Broad lentiviral open reading frame (ORF) collection (housed at UNC). Human ARMC3, CTAGE1, CXORF48, DDX53, DPPA2, FAM46D, GAGE1, IGF2BP3, IGSF11, IL13RA2, LDHC, MAGEA4, MAGEA5, MORC1, PAGE4, PIWIL2, PRAME, SPANXC, XAGE2 and ZNF165 were obtained in pDONR223 and cloned into pLX302 using the GatewayCloning system (Thermo Fisher Scientific). SPANXA2 and XAGE1B cDNAs were obtained in pLX304 (Thermo Fisher Scientific). pCMV6-myc-ZNF165 was obtained from Origene (RC205600). Full-length FATE1 cDNA in pRK5 (a gift from Michael White, UTSW) was subcloned into pCMV-myc (Clontech) and pcDNA3 (Thermo Fisher Scientific) between SalI and NotI, and EcoRI and NotI, respectively. Myc-FATE1 cDNA was from pCMV-myc-FATE was subcloned into pLPCX (Clontech) between BglII and NotI. Full-length RNF183 cDNA was obtained in pLX304 and cloned into pCMV-HA (Clontech) between SalI and NotI. Full-length BIK cDNA was obtained from pEGFP-BIK (Addgene plasmid #10952). HA-RNF183-C13A/C59A and BIK-L61G, which has limited toxicity as compared with wild type^20^, were generated using site-directed mutagenesis. Bcl-xL cDNA (a gift from Mohanish Deshmukh, UNC) was cloned into pCMV-myc between SalI and NotI and then myc-Bcl-xL cDNA was subcloned into pLPCX between BglII and NotI. WISP1 (−1 kb) promoter luciferase construct was in pLightSwitch (Switchgear Genomics). eGFP cDNA (a gift from Michael White, UTSW) was subcloned into pLPCX between BglII and NotI. Viral packaging plasmids, pCMV-dr8.91 and pCMV-VSV-G, were a gift from William Hahn (Harvard). For reporter assays using stable expression, the following expression plasmids were used: TGFβ: pSBE; WNT: pBAR (both gifts from Ben Major, the University of North Carolina at Chapel Hill)^50^; and NF-κB: pGreenFire1-NF-κB (SBI System Biosciences). For transient assays the following reporters were used: TGFβ: pTL-Luc.SMAD (LR0072, Affymetrix); Wnt: M50 Super 8X TOPflash in the pTA-Luc backbone (Addgene plasmid #12456); HIF: pGL3-EPO-HRE3-SV40-LUC^51^ (a gift from David Siderovski, West Virginia University) and pRL-CMV (a gift from Deborah Chapman, the University of Pittsburgh). For ZNF165 shRNA, ZNF165-TRIPz-TetO-shRNA clones V3THS_366604 and V3THS_366599 and TRIPZ-Non-Silencing Control (RHS4743) were purchased from GE Healthcare Life Sciences.

### CTA expression analysis and testbed assembly

For CTA expression analysis, a multiplexed nCounter Gene Expression Codeset (NanoString Technologies) was designed. CTA families with highly similar sequences, including GAGE, SPANX, POTE, CTA45, CTA47, TSPY, MAGEA3&6 and XAGE were assessed by a single expression probe. About 250 ng of mRNA from each cell line was used. Based on NanoString expression values in 19 cell lines, an 11-cell line test bed was identified, which exhibited maximum representation of target CTAs. NanoString probes returning ambiguous expression values (defined as <1,000 counts in all settings) were further evaluated by qPCR Taqman Real-Time PCR Assays (see below for methods). Genes with Ct values <35 were considered expressed.

### Whole genome expression analysis

Triplicate microarray analysis of SUM159 and WHIM12 cells depleted of ZNF165 for 60 h and 48 h, respectively, was performed at the Functional Genomics Core (UNC) on Human GeneChip 1.0 ST Arrays version 1.1 (Affymetrix). Microarray analysis was performed on 250 ng of RNA isolated and terminally labelled with the Ambion WT Expression Kit (Thermo Fisher Scientific). Raw data were normalized using robust multi-array average and significant analysis of microarrays analysis identified significantly modulated genes. Data sets were deposited at the NCBI Gene Expression Omnibus, accession number GSE63986.

### siRNA screen and data processing

Transfection conditions for each cell line were optimized using the CTG assay and the formula: Transfection Efficiency=1−(Luminescence^siUBB^/Luminescence^siCTRL^). A custom siGenome SMART pool siRNA library (Dharmacon/GE Healthcare Life Sciences) was purchased in 96-well plate format and resuspended as described^9^. siRNAs were diluted to 250 nM in serum-free medium and 30 μl of this solution (8.3 pmol of siRNA) was mixed in well with appropriate transfection reagents in 9.8 μl Opti-MEM and incubated for 20 min. Then, 60 or 80 μl of cells in growth medium were added for cell biological and signalling assays, respectively. Cell biological screens were performed 96 h post plating using 20 μl CTG (measuring cellular ATP; viability), 90 μl APO (caspase-3/7 activity; survival) or the Click-iT EdU Alexa Fluor 488 Imaging Kit (DNA synthesis; proliferation) assay systems according to manufacturer's protocols. CTG and APO assays were read with a Pherastar Plus or Pherastar FS (BMG Labtech) plate reader and EdU incorporation imaged using a Cellomics ArrayScan HCS Reader (Thermo Fisher Scientific). Incorporation was calculated as indicated by EdU-positive nuclei/total nuclei via staining with Hoechst 3342, trihydrochloride and trihydrate. For the HIF screen, 100 ng of pGL3-EPO-HRE3-SV40-LUC, 2 ng of pRL and 8.3 pmol of siRNA were reversed transfected with DharmFECT Duo. Cells were treated with 1 mM DMOG or vehicle 32 h after transfection. After an additional 16 h, firefly and renilla luciferase levels were quantitated with Dual Glo Luciferase Assay System modified to use 20 μl of both the Luciferase and Stop and Glo reagents per well. For other signalling assays, 44 h post transfection, cells were exposed to vehicle or ligand for 16 h and luciferase activity measured using the ONE-Glo Luciferase Assay System modified to use 20 μl of OneGlo reagent per well. All luminescence assays were measured on a PHERAstar Plus. Raw values for each cell biological screen were normalized to a non-targeting control (siGENOME Non-Targeting siRNA Pool#2 (GE Healthcare Life Sciences)). Raw data were first normalized to CTG values taken in parallel to correct for viability defects (with the exception of the HIF screen, which used renilla luminescence values) and then *z*-scores were calculated. siRNA pools with *z*-scores >2 in the apoptosis screen or <−2 in the viability and proliferation screens were considered outliers. Only siRNA pools that exhibited statistically significant change (*P*≤0.05 by unpaired Student's *t*-test) were considered. For the HIF, NF-κB, Wnt and TGFβ reporter screens, siRNA pools that reduced reporter activity by either >60% in a single cell line or >30% in more than two cell lines were considered positives. For the NF-κB, Wnt and TGFβ signalling screens, both basal- and ligand-induced values were considered.

### Transfections

For siRNA transfection, cells were trypsinized and plated into medium containing 50–100 nM siRNA complexed with RNAiMAX in Opti-MEM and incubated as indicated in the figure legends. For cDNA transfections, HeLa, H1155 and H1299 cells were transfected using Lipofectamine 2000 (Thermo Fisher Scientific) and HEK293T cells were transfected using FuGENE 6 or the calcium phosphate method^52^. All manufacturers' protocols were followed.

### Generation of stable cell lines

Cell lines stably expressing luciferase reporters and WHIM12-HcRed and WHIM12-ZNF165-V5 cell lines were created via lentiviral-mediated gene transduction through co-transfection of HEK293T cells with viral expression and packing plasmids (pCMV-VSV-G and pCMV-dr8.91). For FATE1 studies, stable lines were created via retroviral-mediated gene transduction through co-transfection of HEK293GP cells with pLPCX expression plasmids and pCMV-VSV-G. Virus conditioned media was used to infect target cells in the presence of polybrene and stable populations were selected using appropriate antibiotics.

### Luciferase assays

Indicated luciferase reporters (100 ng), Renilla reporter (pRL-CMV, 2 ng) and 100 ng indicated cDNAs were transfected into HEK293T using Fugene 6. Forty-eight hours later, luciferase activity was measured using the Dual Glo Luciferase Assay System. For WISP1 promoter luciferase assay, 100 ng of the pLightSwitch-WISP1(−1 kb) promoter was used and luciferase activity was quantitated with Renilla Luciferase Assay System (Promega). Luciferase values were normalized to CTG in parallel samples.

### Colony formation assay

At indicated incubation times following siRNA transfection, cells were replated at limiting dilution, fed every other day and stained with Geimsa (Sigma).

### Gene expression

RNA was isolated using TRIzol or an RNA isolation kit (Sigma) and reverse transcribed using the High-Capacity cDNA Reverse Transfection Kit (Thermo Fisher Scientific) according to manufacturer's instructions. An Applied Biosystems Real-Time PCR system and either Solaris (Dharmacon), SYBR Green or TaqMan Real-Time PCR (Thermo Fisher Scientific) gene expression assays were used. Gene expression assays were multiplexed with RPL27, GAPDH or actin as control assays. Relative expression values were calculated using the comparative 2^−ΔΔCT^ method^53^. Primer sequences or catalogue numbers can be found in Supplementary Data 7.

### Immunoblotting

Whole cell lysates were prepared in 2 × Laemmli sample buffer and resolved using SDS–PAGE. Gels were transferred to Immobilon PVDF (Millipore) or nitrocellulose (Bio-Rad Laboratories) membranes, blocked in either tris-buffered saline containing 0.1% Tween-20 (TBST) and 5% non-fat dry milk or 5% bovine serum albumin (BSA), or Odyssey (LI-COR Biosciences) blocking buffer followed by incubation with indicated primary antibodies for 1 h or overnight. After washes in TBST, appropriate horseradish peroxidase-coupled secondary antibodies (Jackson Immunoresearch) or IRDye antibodies (LI-COR Bioscience) were used for chemiluminescence or fluorescence detection (Odyssey), respectively. Whole immunoblots are provided in Supplementary Figs 5–10.

### Immunofluorescence

Cells plated on glass coverslips were fixed with 3.7% formaldehyde and then permeabilized with 0.5% Triton X-100 for 10 min. For calnexin staining, cells were permeabilized with ice-cold methanol for 10 min. Cells were blocked and washed in 1% BSA, 0.1% Tween-20 in 1 × phosphate buffered saline (PBS) (PBTA). Cells were incubated with primary antibodies for 1 h followed by three washes in PBTA. Coverslips were then incubated with Alexa Fluor-conjugated secondary antibodies (Thermo Fisher Scientific) for 30 min followed by three washes in PBTA and a wash in H_2_O. MitoTracker was added for 30 min prior to fixation. Prolong Gold Antifade reagent with DAPI (Thermo Fisher Scientific) was used to mount slips on glass slides and images were acquired on either a Leica DM55000 B upright microscope, a Leica TCS SP5 confocal microscope, a Zeiss Axioimager upright microscope or a Zeiss LSM510 confocal microscope.

### Viability assays

Cells were reverse transfected with RNAiMAX in Opti-MEM with 50–100 nM siRNA in 96-well format. In TNBC assays, cells were fed at 48 h. About 96 h post transfection (unless otherwise indicated), CTG was used to quantitate total ATP using a Pherastar Plus plate reader.

### Immunoprecipitation

Unless otherwise indicated, cells where lysed on ice for 30 min in non-denaturing lysis buffer (NDLB): 50 mM HEPES pH 7.4, 1.0% Triton X-100, 0.5% sodium deoxycholate, 150 mM NaCl, 1 mM NaVO_4_, 25 mM β-Glycerophosphate, 1 mM ethylenediaminetetraacetic Acid (EDTA), 1 mM ethylene glycol tetraacetic acid (EGTA), 1 μg ml^−1^ pepstatin, 2 μg ml^−1^ leupeptin, 2 μg ml^−1^ aprotinin and 50 μM bestatin. Lysates were clarified at 12,000*g* for 10 min. Then, 10% of each clarified lysate was set aside as an input loading control and the remainder was immunoprecipitated for 4 h at 4 °C with antibodies coupled to Protein A/G beads. Unless otherwise indicated, beads were washed two times in high salt (350 mM NaCl) NDLB, once in NDLB and then resuspended and boiled in 2 × Laemmli sample buffer.

### Autoubiquitination assays

For *in vitro* autoubiquitination assays, HEK293T were transfected with pCMV-HA-RNF183 or pCMV-HA-RNF183-CC/AA. Twenty-four hours after transfection, cells were lysed on ice in NDLB, pH=8.0, clarified at 12,000*g* for 10 min and then immunoprecipitated for 4 h with anti-HA antibody (Covance) and Protein A/G beads (Life Technologies). Beads were then washed three times in NDLB with 350 mM NaCl and two times in ligase buffer: 50 mM Tris pH 7.5, 150 mM KCl, 1 mM MgCl2. After the final wash, beads were resuspended in ligase buffer containing 100 nM recombinant E1 (Enzo Life Sciences), 1 μM recombinant UbcH5b (Enzo Life Sciences), 5 μM ubiquitin from bovine erythrocytes (Sigma), plus or minus 5 mM Mg2+ ATP and incubated for 1.5 h at 37 °C.

### ChIP and ChIP-Seq

WHIM12-ZNF165-V5 cells were grown to 75% confluency and cross-linked with 1% formaldehyde for 10 min at 25 °C. Crosslinking was quenched with 125 mM glycine. Nuclei were isolated by dounce homogenization in hypotonic buffer (20 mM HEPES pH 7.9, 10 mM KCl, 1 mM EDTA, 10% glycerol, 1 μg ml^−1^ pepstatin, 2 μg ml^−1^ leupeptin, 2 μg ml^−1^ aprotinin and 50 μM bestatin) followed by centrifugation at 600*g* for 5 min and then lysed in RIPA buffer (10 mM Tris-HCl, pH 7.5, 150 mM NaCl, 1.0 mM EDTA, 1.0% Sodium Deoxycholate, 0.1% SDS, 1.0% NP-40, 0.2 mM NaVO_4_, 1 μg ml^−1^ pepstatin, 2 μg ml^−1^ leupeptin, 2 μg ml^−1^ aprotinin and 50 μM bestatin). DNA was sheared to a range of 300–500-bp fragments. Chromatin was immunoprecipitated using 2 μg of ChIP-Grade anti-V5 (Abcam ab9116) overnight at 4 °C followed by a 2-h incubation with Protein A/G beads. ChIPed DNA was recovered by reverse crosslinking with an overnight incubation at 65 °C. Excess RNA and protein were removed, respectively, with 100 μg RNAse and 10 μg Proteinase K, and the remaining DNA was purified using the Zymogen Zymo-Spin ChIP-Grade DNA Clean-Up Kit.

For sequencing of chromatin immunoprecipited DNA, 5 ng of immunoprecipitated DNA underwent library preparation using the KAPA HTP Library Preparation Kit. Sequencing was performed on the Illumina HiSeq 2500 using 50SR V3 reagents. Following sequencing, HOMER findPEAKS module was used to identify significantly enriched peaks using a false discovery rate cutoff ≤0.001, a cumulative Poisson *P* value and required fourfold or more enrichment of the ratio of normalized sequence reads in the experimental sample to the normalized sequences reads in the input sample. GREAT was used to assign peak regions to genes and evaluate pathway enrichment^54^,^55^. HOMER package v4.2 findmotifsGenome.pl module with settings: −size150 −S 10-bits^56^ was used for *de novo* motif analysis. Motif analysis was restricted to 150 bp surrounding peak summit. ChIP-Seq data sets were deposited at the NCBI Gene Expression Omnibus, accession number GSE63986.

### ChIP–qPCR analysis

ChIP–qPCR analysis was conducted using custom SYBR Green assays designed to ChIP-Seq peaks (See Supplementary Data 7). The ACRBP ORF lacked enrichment in the WHIM12-ZNF165-V5 ChIP-Seq and was used as a negative control for ZNF165-V5 binding. ZNF165-V5 occupancy was evaluated with immunoprecipitation efficiency using per cent of total input DNA of WHIM12-CTRL and WHIM12-ZNF165-V5 cells.

### Xenograft injections

WHIM12 cells (650,000) stably expressing ZNF165-TRIPz-TetO-shRNA or a non-targeting control hairpin in 200 μl PBS were injected in the right flank of 4–6-week-old female NOD.cg-PRKDC^SCID^Il2rg^tm1Wjl^/SzJ (NSG) mice. Twenty-four hours prior to injection, cells were exposed to 1 μg ml^−1^ doxycycline and mice were administered water containing 2 mg ml^−1^ doxycycline and 1% sucrose *ad libitum*. Tumours were measured twice weekly using a digital caliper and volume (*V*) was calculated using the formula: *V*=(Longest side)(perpendicular to longest side)^2^/(*π*/6). Tumours were surgically removed, weighed, formalin fixed for 48 h, sectioned (5 μm) and immuonostained with Ki-67. Ki-67 fluorescence (Fluorescence^Total^−Fluorescence^Background^) was calculated using the ImageJ integrated density function. All studies were conducted in accordance with a UTSW Institutional Animal Care and Use Committee (IACUC) approved protocol.

### Human breast tissue

All human breast tissue was obtained from the UNC Lineberger Tissue Procurement center in compliance with guidelines of the UNC Internal Review Board committee. All human tissues were obtained with informed consent. Samples were homogenized in lysis buffer (1% Triton X-100, 50 mM HEPES pH 7.6, 150 mM NaCl, 1.5 mM MgCl_2_, 1 mM EGTA, 25 mM β-glycerolphosphate, 10% Glycerol, 1 mM EGTA, 1 mM NaVO_4_, 1 μg ml^−1^ pepstatin, 2 μg ml^−1^ leupeptin, 2 μg ml^−1^ aprotinin and 50 μM bestatin). Samples were sonicated and the soluble fraction recovered by centrifugation. Protein concentrations were quantitated using Bio-Rad Protein Assay Dye Reagent. Samples were diluted to 1 mg ml^−1^ in 4 × Laemmli sample buffer and immunoblotted as described above. Coomassie stain (Genlantis) was incubated with SDS–PAGE gels for 30 min followed by destain for 4 h.

### Oncomine analysis

For Oncomine BIK mRNA tumour/normal analysis, the following studies were used: GSE16515, GSE2514 and TCGA Research Network (http://cancergenome.nih.gov/)^57^,^58^,^59^,^60^. For Oncomine ZNF165 mRNA tumour/normal analysis, the following studies were used: GSE4336, GSE6764, GSE12470 and GSE7410 (refs 61, 62, 63, 64).

### Survival analysis

The overall survival and clinicopathological data sets were GSE8894, Lung Adenocarcinoma and Colorectal TCGA (downloaded from cBioPortal)^60^,^65^, Molecular Taxonomy of Breast Cancer International Consortium (METABRIC) and GSE42127, the latter was supplemented to 209 cases^66^. Samples were placed into groups based on a median cutoff, with the exception of the colorectal patients in Fig. 3j, where a *z*-score cutoff of expression >1.5 was used and METABRIC patients where the gene expression low and high cutoff point was determined by the mclust R version 4 from the R package^67^. The distributions of time-to-event outcomes were estimated using the method of Kaplan and Meier. *P* values and HRs were calculated using a Cox regression model.

## Additional information

**Accession codes**: The microarray and ChIP-Seq data have been deposited in the Gene Expression Omnibus under accession code GSE63986.

**How to cite this article:** Maxfield, K. E. *et al.* Comprehensive functional characterization of cancer–testis antigens defines obligate participation in multiple hallmarks of cancer. *Nat. Commun.* 6:8840 doi: 10.1038/ncomms9840 (2015).

## Supplementary Material

Supplementary Information Supplementary Figures 1-10 and Supplementary References

Supplementary Data 1 Description of CT-antigens screened.

Supplementary Data 2 Definition of CTA Expression. (a) Nanostring Codeset with sequences for detection of each gene. (b) Nanostring expression values across twenty tumor derived cell lines. (c) Nanostring and qPCR Ct values for CT-antigens across testbed cell lines.

Supplementary Data 3 Z-scores for (a-c) cell biological screen and (d-g) ligand-induced signaling screens.

Supplementary Data 4 (a-d) CTA's scoring in signaling screens (ligand induced or basal signaling).

Supplementary Data 5 WHIM12-ZNF165-V5 ChIP-Seq. (a)WHIM12-ZNF165-V5 ChIP-Seq peaks and GREAT-associated genes. (b) GREAT-associated genes that comprise the enriched pathways generated from GREAT Pathway Analysis.

Supplementary Data 6 Global Modulation of TGFβ-responsive genes by ZNF165. (a) Fold change, FDR and T-test for all SUM159 +/- TGFβ-induced genes +/- siZNF165. (b) All significantly modulated following ZNF165 depletion across entire Affymetrix microarray regardless of TGFβ stimulation in SUM159 cells. (c) Fold change, FDR and T-test for all WHIM12 +/- TGFβ-induced genes +/- siZNF165. (d) All significantly modulated following ZNF165 depletion across entire Affymetrix microarray regardless of TGFβ stimulation in WHIM12 cells.

Supplementary Data 7 Table containing catalog number or primer sequences for qPCR primers used in this study.
