# Supplementary material for: Comprehensive functional characterization of cancer–testis antigens defines obligate participation in multiple hallmarks of cancer
Source: Nat Commun. 2015 Nov 16;6:8840. doi: 10.1038/ncomms9840 (PMC4660212; doi:10.1038/ncomms9840)
Supplement: Supplementary Information — Supplementary Figures 1-10 and Supplementary References [file ncomms9840-s1.pdf]

a.

	Cell Biological Screening Platform		
Phenotype	Viability	Apoptosis	Proliferation
Assay	CellTiter-Glo™	Apo-ONE™	DNA Synthesis/ EdU Incorporation
Time point (hours)	96	96	96
Model	SK-OV-3, SK-OV-6, SK-MEL-37, SK-MEL-2, Saos-2, H460, H1299, HCC4017, WHIM12, MCF-7, LNCaP	SK-OV-3, SK-OV-6, SK-MEL-37, SK-MEL-2, Saos-2, H460, H1299, HCC4017, WHIM12, MCF-7, LNCaP	SK-OV-3, SK-OV-6, SK-MEL-37, SK-MEL-2, Saos-2, H460, H1299, HCC4017, WHIM12, MCF-7, LNCaP

	Signaling Pathway/ Luciferase Based Reporter Platform			
Signaling Pathway	Wnt Signaling	TGFβ Signaling	NF-κB Signaling	HIF Signaling
Assay	Wnt-3A induced transcription	TGFβ induced transcription	TNFα induced transcription	DMOG induced transcription
Time point (hours)	60	60	60	48
Model	SK-OV-3, SK-OV-6, SK-MEL-37, SK-MEL-2, Saos-2, H460, H1299, HCC4017, WHIM12	SK-OV-3, SK-OV-6, SK-MEL-37, SK-MEL-2, H1299,HCC4017, WHIM12	SK-MEL-37, SK-MEL-2, H1299, WHIM12, LNCaP	SK-OV-3,SK-MEL-2 SK-OV-6,SK-MEL-37, LNCaP, H1299,Saos-2, HCC4017, WHIM12
Ligand Dose	500 ng/ml; 16 hrs	10 ng/ml; 16 hrs	25 ng/ml; 16 hrs	1 uM; 16 hrs

c.

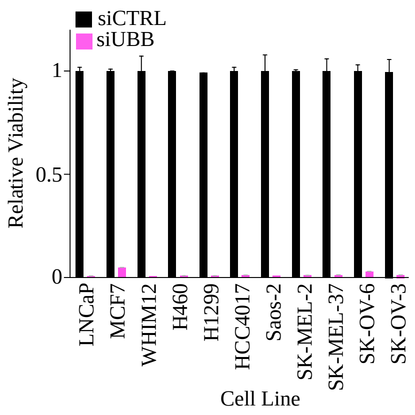

b.

	Optimized Cell Densities			Transfection Reagent Volume (uL)		
				Cell Biological Screen and Stable Reporter Assays		Transient Reporter
Cell Line	Biological Screen	Lenti-Transduced Reporter Assays	Transient Reporter Assay (HIF)	RNAiMAX	DharmaFECT1	DharmaFECT Duo (HIF)
LNCaP	10K	10K	25K	0.2	-	0.2
MCF7	9K	10K	-	0.2	-	0.2
WHIM12	2K	4K	15K	0.2	-	0.2
HCC4017	4K	6K	18K	0.2	-	0.4
H1299	2K	7K	9K	0.2	-	0.2
H460	5K	5K	-	-	0.2	0.2
SK-MEL-37	5K	5K	15K	0.2	-	0.2
SK-MEL-2	10K	10K	27K	0.2	-	0.2
Saos-2	5K	5K	25K	0.2	-	0.2
SK-OV-3	3K	8K	22K	0.2	-	0.2
SK-OV-6	5K	5K	15K	0.2	-	0.6

d.

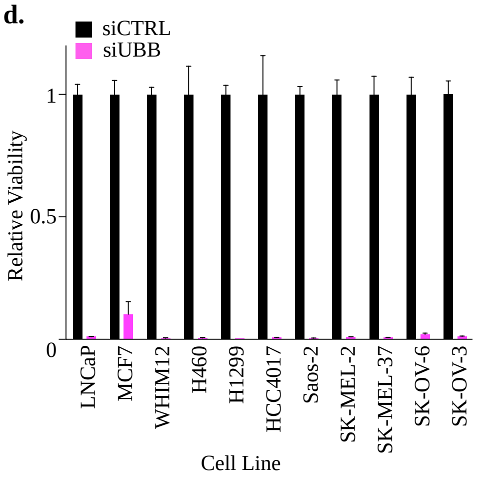

e.

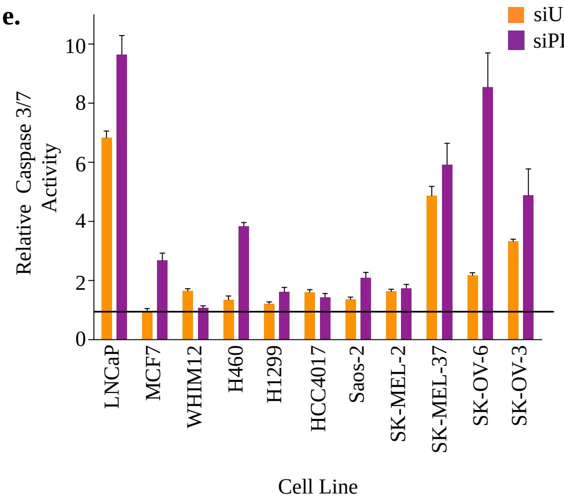

f.

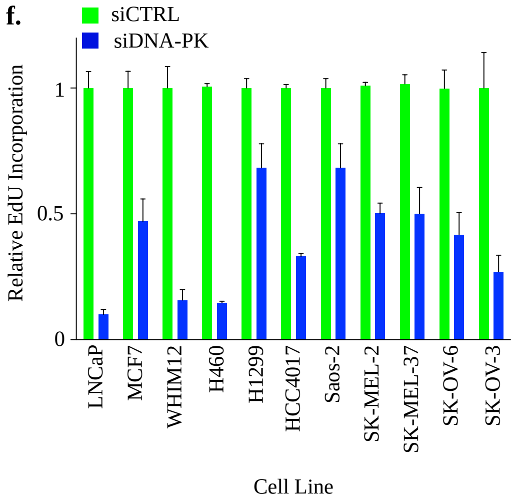

Supplementary Figure 1, Related to Figure 1. (a) Parameters for screens. (b) Plating densities and transfection reagent conditions for each cell line in each screen. (c) Transfection efficiency in testbed cell lines. Cell viability was measured in each cell line by CTG 96 hours after transfection with a non-targeting control (siCTRL) or UBB siRNA. siUBB values are normalized to siCTRL. Bars represent mean ($n = 2$) ± range. (d-f) Graph of positive controls (siUBB, siPLK1 and siDNA-PK) values normalized to negative control for viability (CTG), apoptosis (APO) and proliferation (EDU) screens, respectively. Bars represent mean ($n \geq 4$) ± standard deviation (s.d.).

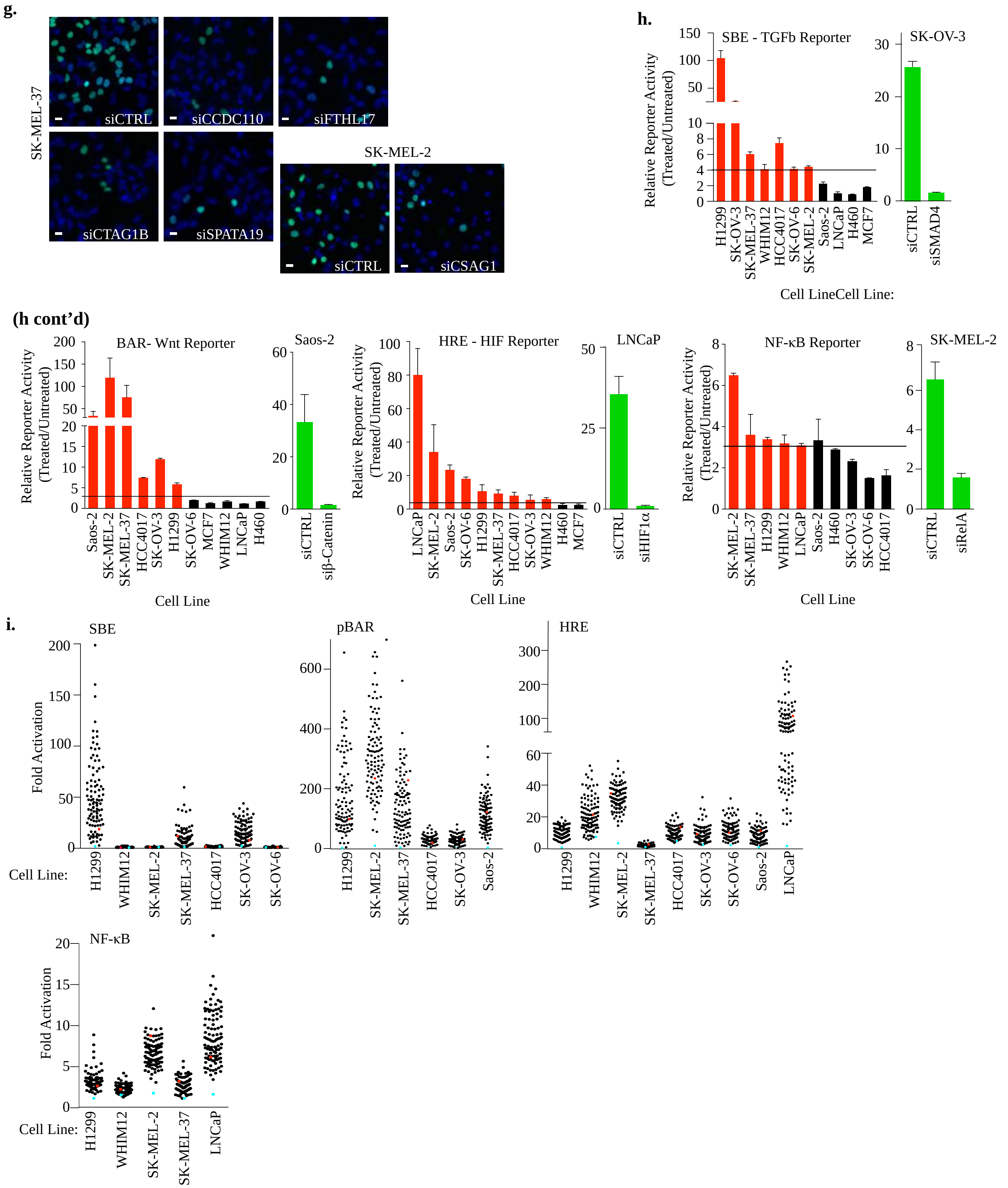

Supplementary Figure 1 continued, Related to Figure 1. (g) Representative raw images of Hoechst and EDU staining from the EdU screen. Blue: Hoechst stain, Green: anti-EdU. Scale bars represent 10 μm. (h) Reporter activity measured following ligand stimulation. Indicated cell lines expressing each reporter were stimulated with ligands (as indicated in (b)) and reporter activity measured as described in the Material and Methods section. Red bars indicate cell lines exhibiting induction sufficient for screening analysis. In green panels, indicated cell lines were transfected with siRNAs targeting indicated transcriptional proteins of each pathway prior to treatment. Bars represent mean ($n \geq 2$) ± range. (i) Distribution of Fold-Activation for each signaling screen. Black dots represent siCTAs, Red are negative controls (siCTRL) and Cyan are positive controls.

a.

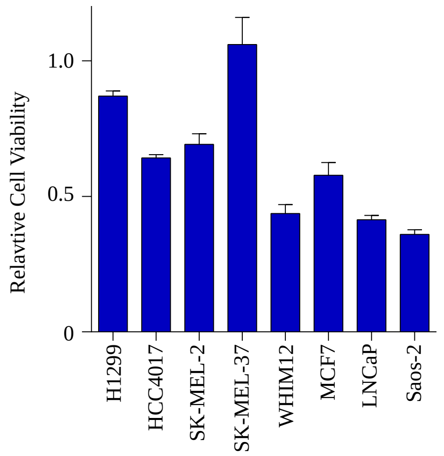

b.

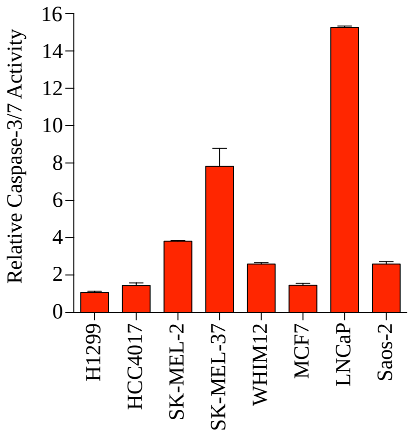

d.

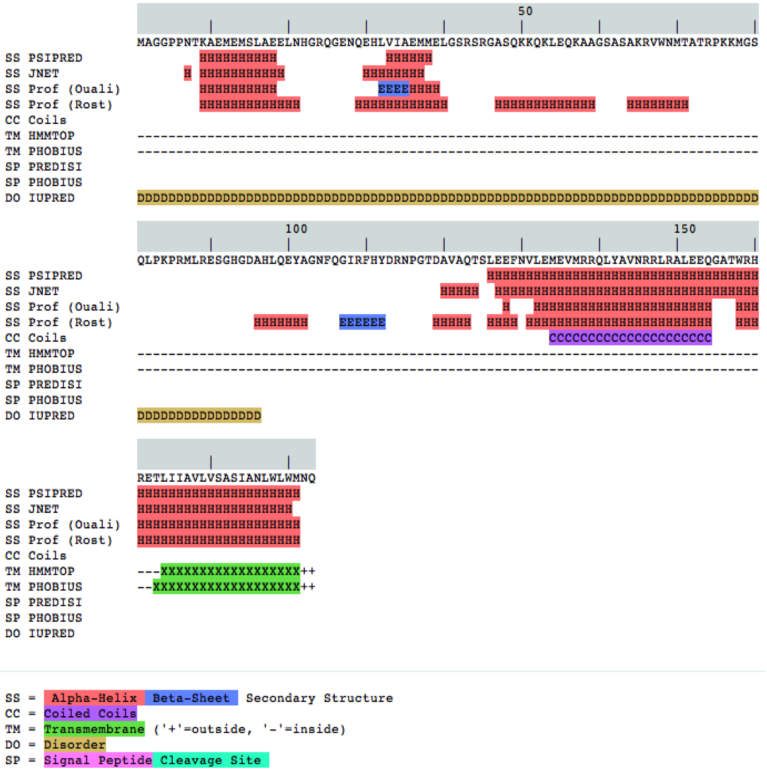

c.

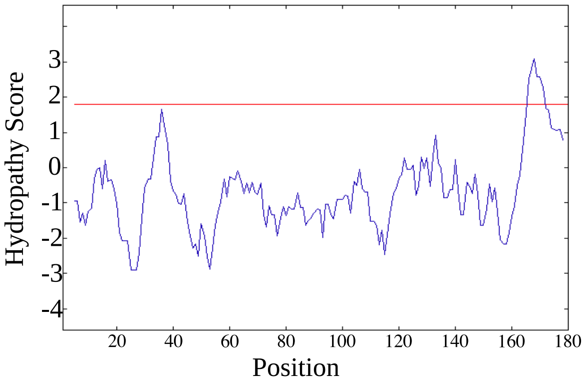

e.

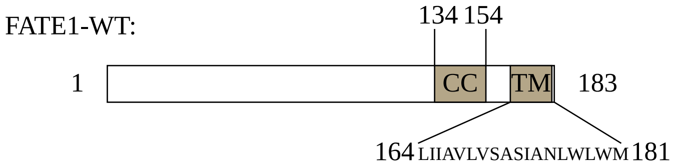

Supplementary Figure 2, Related to Figure 2. (a) Viability assay (relative to non-targeting control) in testbed cell lines exhibiting FATE1 expression 96 hours after siFATE1 transfection. Bars represent mean (n ≥ 2) ± range. (b) Relative (to non-targeting control) Cleaved Casapse 3/7 activity in testbed cell lines exhibiting detectable FATE1 expression 96 hours after siFATE1 transfection. Adapted from screening anlaysis. Bars represent mean (n = 3) ± s.d. (c) Kyte-Doolittle Hydropathy Plot of FATE1 amino acid sequence calculated with a window size of 9. Red line indicates cut off for possible transmembrane regions. (d) Quick2D protein motif output for FATE1 amino acid sequence. (e) Domain map of FATE1. CC: Coiled Coil; TM: Transmembrane.

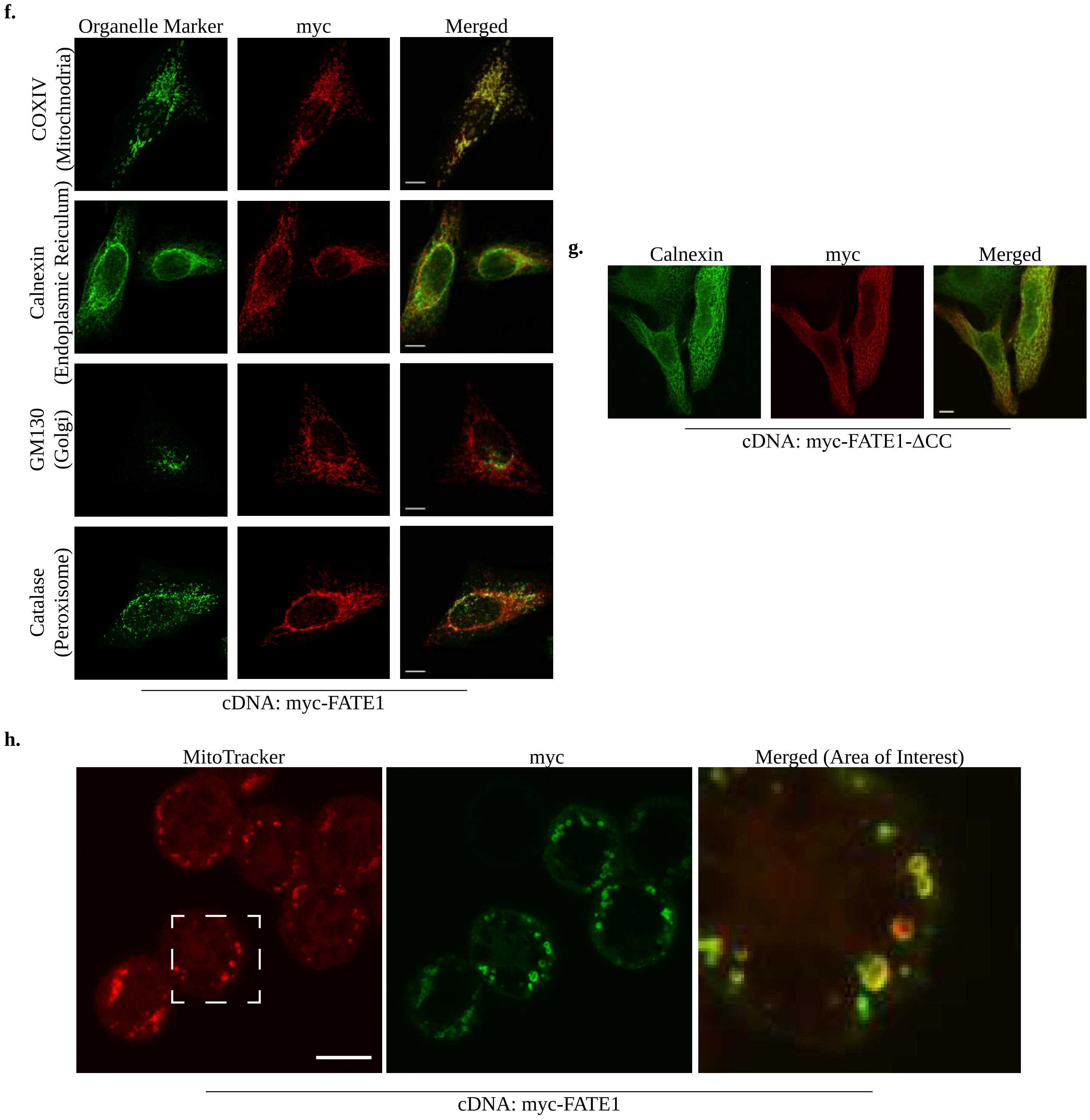

Supplementary Figure 2 continued, Related to Figure 2. (f and g) HeLa cells transfected with myc-FATE1 for 24 hours were fixed, stained with indicated antibodies, and imaged with confocal microscopy. Data representative of 2 independent assays. Scale bars represent 10 µm. Organelles (left) are in green, myc-FATE1 is in red. (h) H1155s transiently transfected with myc-FATE1 cDNA were incubated with MitoTracker (red) for 30 minutes, fixed, stained with anti-myc (green), and imaged using confocal microscopy. Data representative of 2 independent assays. Scale bar represents 10 µm.

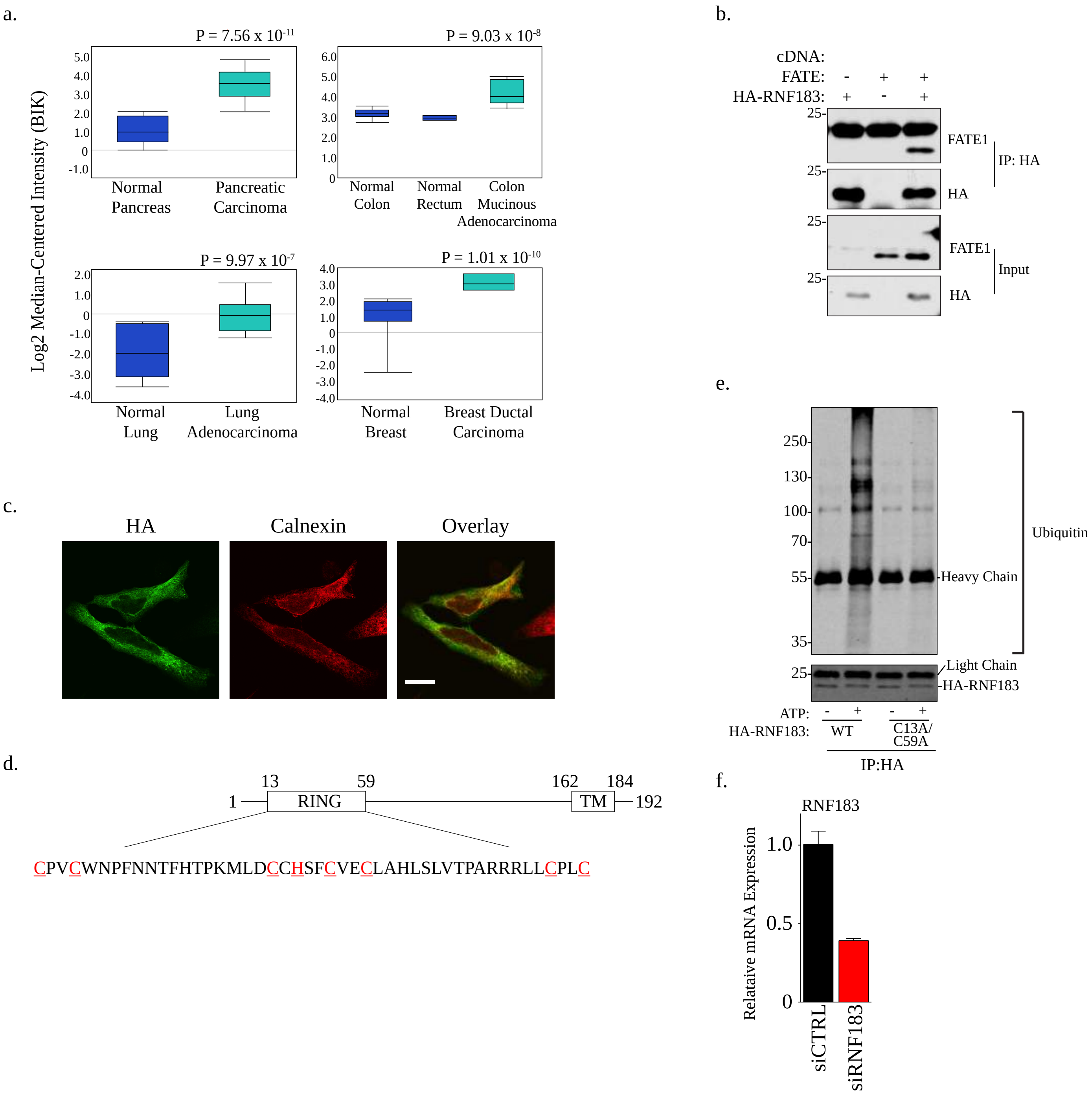

Supplementary Figure 3, Related to Figure 3. (a) Expression of BIK mRNA in indicated tumor tissues[1-4]. Graphs generated by Oncomine™. (b) HEK293T cells were transfected with indicated plasmids for 24 hours, lysates prepared (pH 8.0) and then immuno-precipitated with anti-HA and immunblotted as indicated. Data are representative of 3 independent assays. (c) Twenty-four hours after transfection with pCMV-HA-RNF183, HeLa cells were fixed, immunostained with indicated antibodies, and imaged using confocal microscopy. Data representative of 2 independent assays. Scale bars represent 10 μm. Green is HA, Calnexin (red) is used to visualize the ER. (d) RNF183 domain structure based on the Human Protein Reference Database (HPRD). Conserved Zn++ -coordinating residues are underlined in red. RING: Really Interesting New Gene; TM: Transmembrane. (e) HEK293T cells transfected with indicated cDNAs for 24 hours were immunoprecipitated and subjected to an autoubiquitination assay as described in Methods. Data representative of 2 independent assays. (f) HCT116 cells were transfected with indicated siRNAs in parallel to Figure 3f. Forty-eight hours after transfection, RNF183 gene expression was measured using qPCR Bars represent mean (n = 2) ± range.

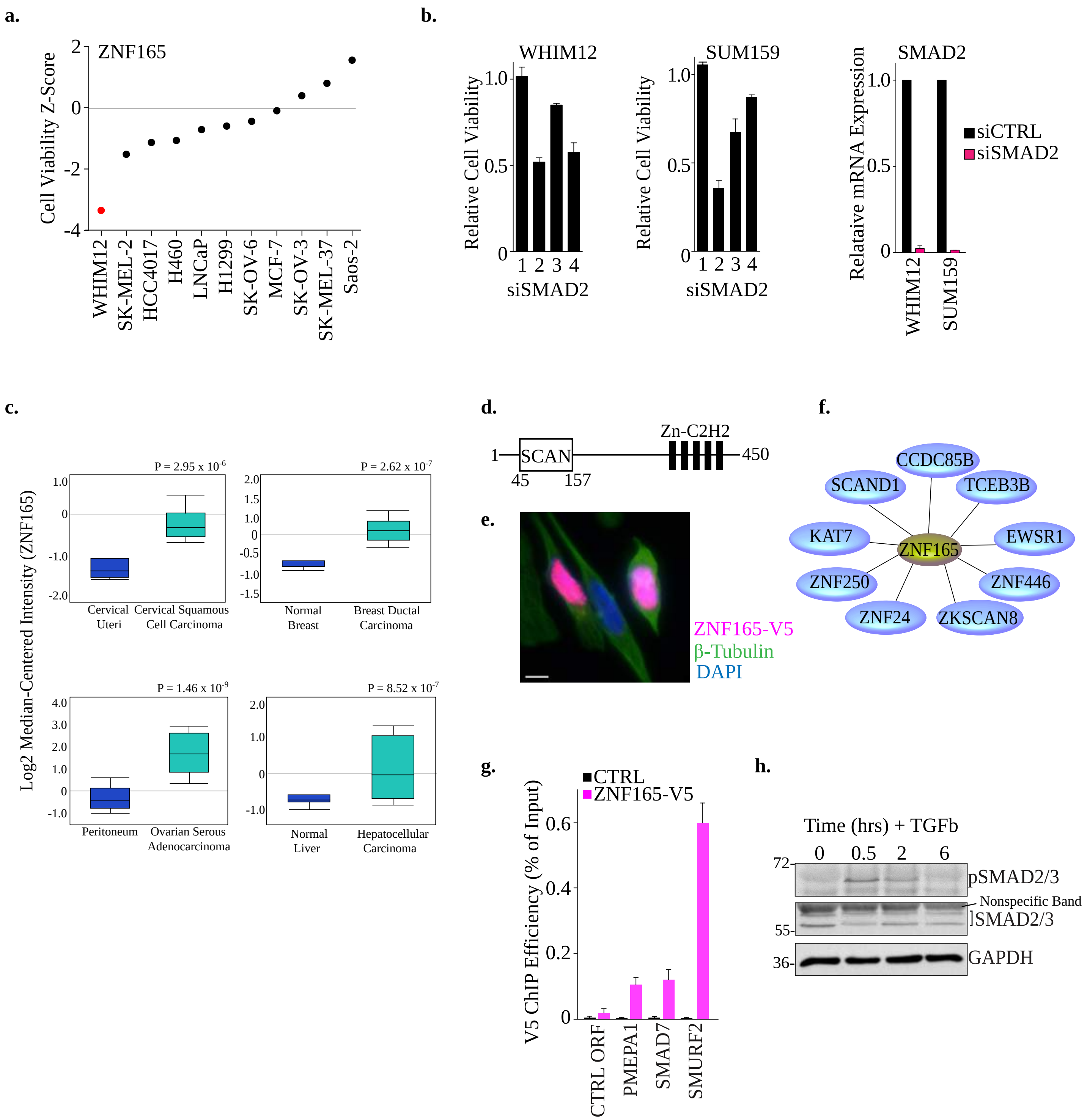

Supplementary Figure 4, Related to Figure 5&6. (a) Z-scores for siZNF165 for indicated cell lines from cell viability screen. Data represents at least 2 independent assays. (b) Left two panels: Normalized cell viability for indicated cell lines transfected with indicated siRNA oligos for 96 hours. Bars represent mean (n ≥ 2) ± range. Right: Relative SMAD2 mRNA expression quantitated 72 hours post transfection with indicated siRNAs. Bars represent mean (n = 2) ± range. (c) ZNF165 mRNA expression in indicated tissues[5-8]. Graphs generated by Oncomine™. (d) Domain map of ZNF165. SCAN: SCAN domain; Zn-C2H2: canonical zinc finger motifs. (e) HeLa cells transfected with ZNF165-V5 for 48 hours were immunostained as indicated. Data are representative of 2 independent assays. Scale bar represents 10 μm. Pink is V5, Green is β-tubulin and blue is DAPI. (f) ZNF165 interactors in yeast two-hybrid proteomics analyses[9,10]. (g) ChIP-qPCR analysis for indicated genes in WHIM12 cells stably expressing ZNF165-V5 or HcRED (CTRL). Bars represent mean percent input (n = 2) ± range. (h) WHIM12 cells were exposed to vehicle or 10 ng/mL TGFβ for indicated times and immunoblotted as indicated.

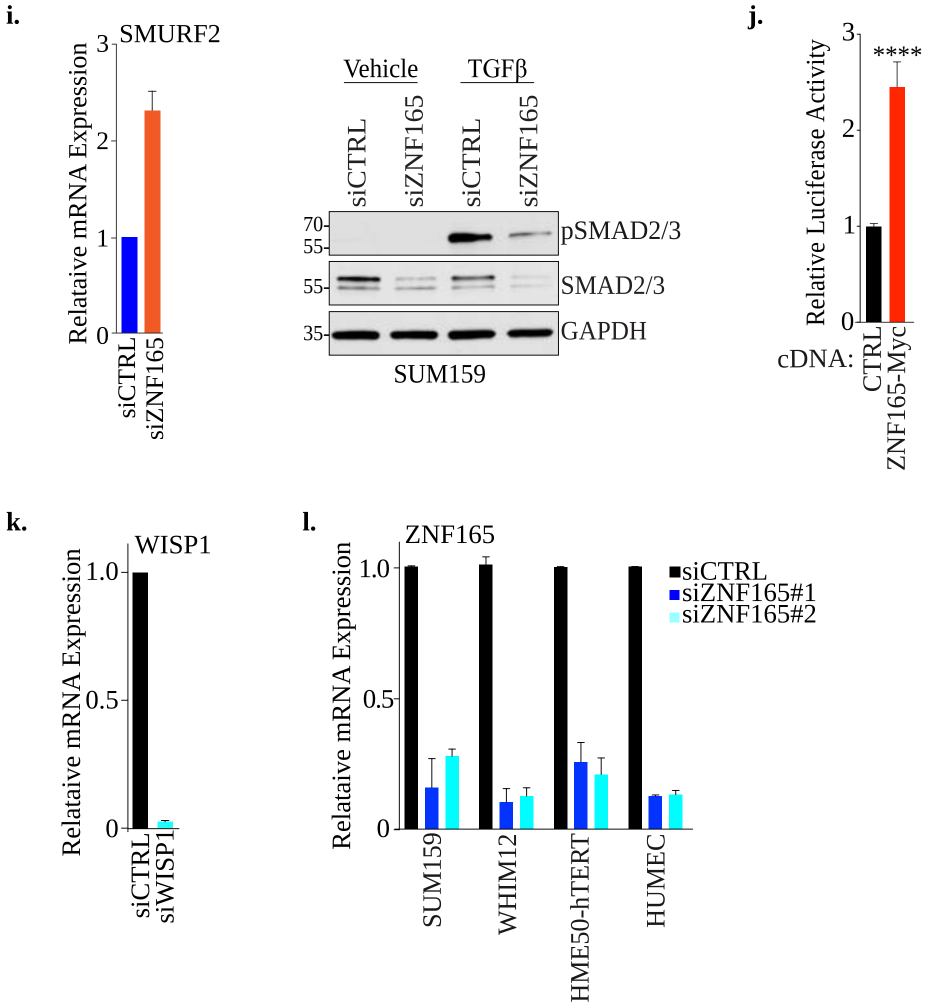

Supplementary Figure 4 continued, Related to Figure 5&6. (i) Left: SUM159 cells were transfected as indicated for 48 hours and relative SMURF2 mRNA expression quantitated using qPCR. Bars represent mean (n = 3) ± range. Right: SUM159 cells were treated as in left panel and exposed to vehicle or 10 ng/mL TGFβ for 30 minutes. Whole cell lysates (WCLs) were immunoblotted as indicated. Data are representative of 3 independent assays. (j) 48 hours post transfection of HEK293T cells with indicated cDNAs and the WISP1 promoter fused to a luciferase reporter, luciferase activity was quantitated. Bars represent mean (n = 16) ± s.e.m. (k) SUM159 cells were transfected with indicated siRNAs for 72 hours followed by quantitation of relative WISP1 mRNA expression using qPCR. Bars represent mean (n ≥ 2) ± range. (l) Relative ZNF165 mRNA expression was quantitated using qPCR following 48 hour transfection with indicated siRNAs in indicated cell lines. Bars represent mean (n = 2) ± range.

Figure 2c

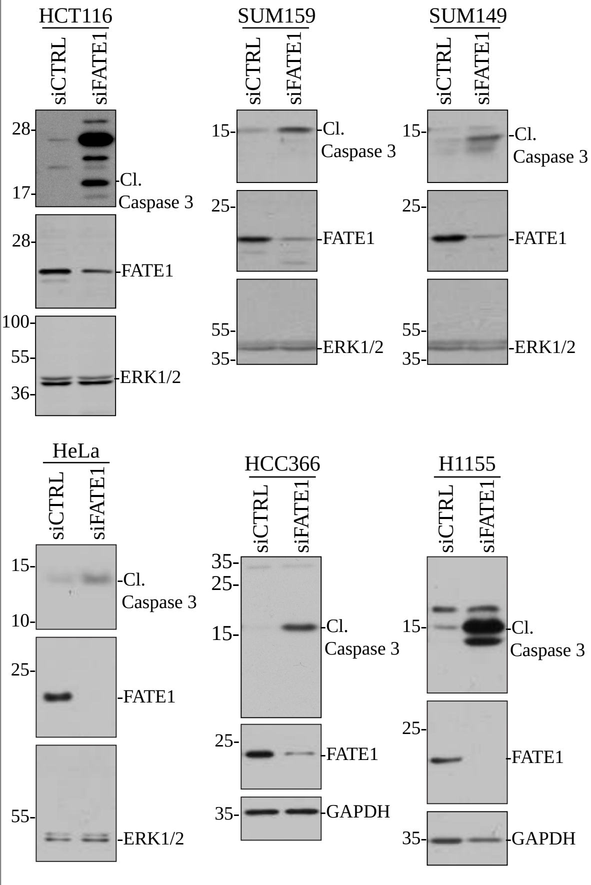

Figure 2e

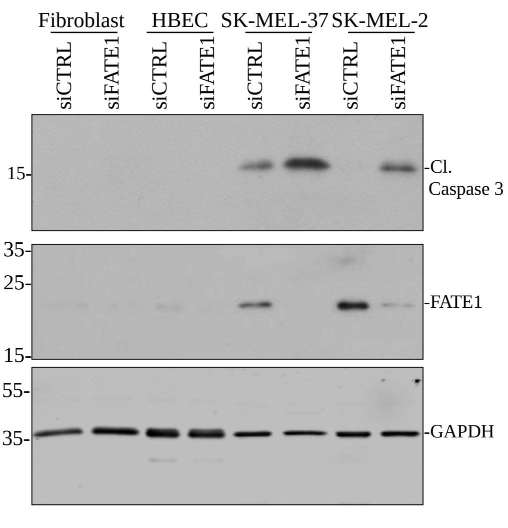

Figure 2g

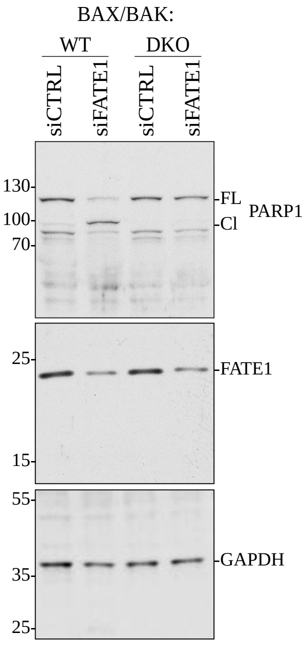

Figure 2h

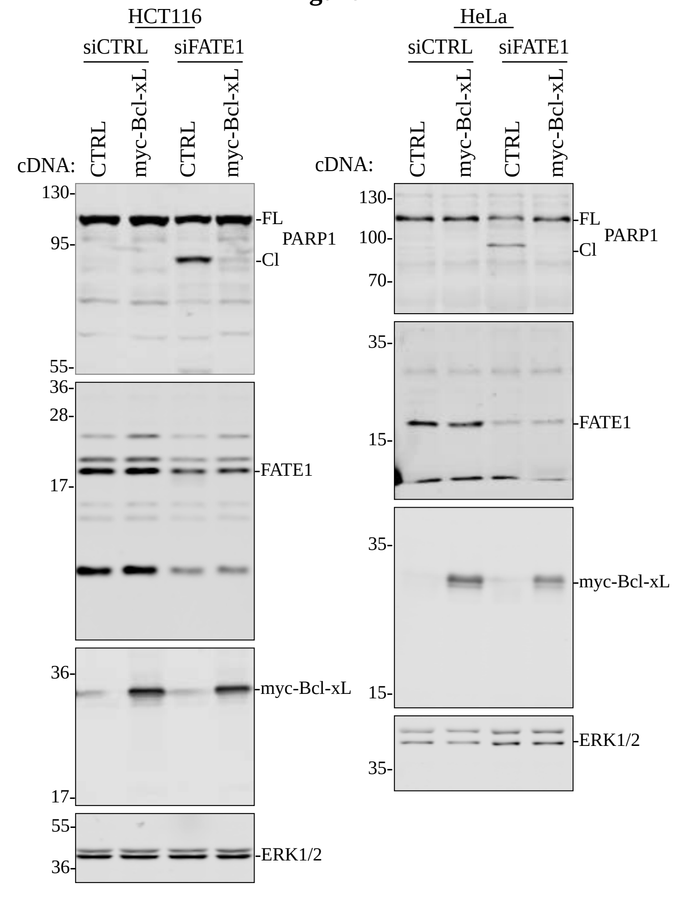

Figure 2i

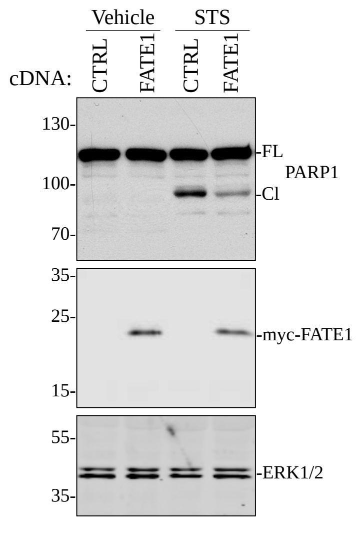

Supplementary Figure 5: Whole blots for Figure 2

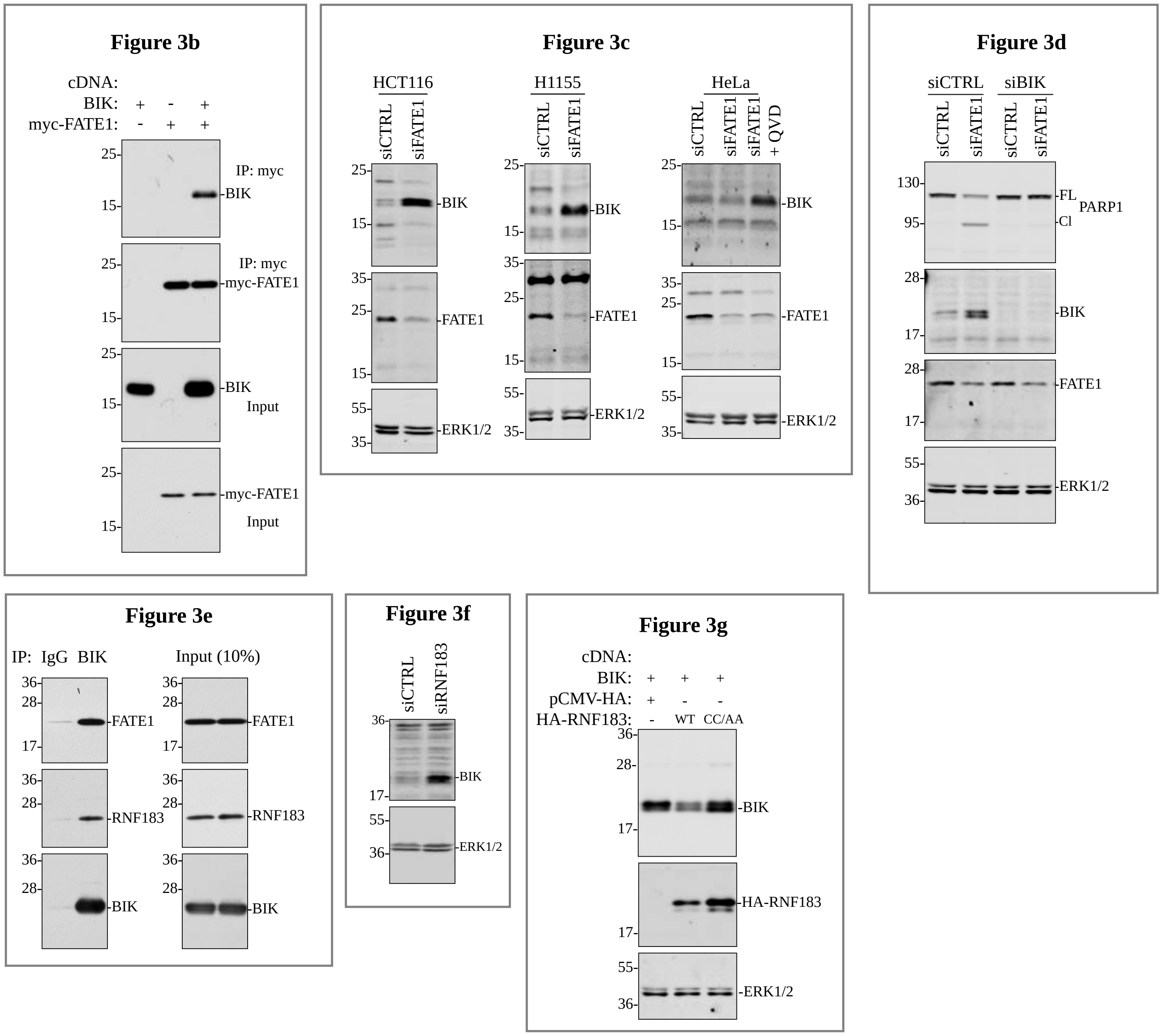

Supplementary Figure 6: Whole blots for Figure 3

Figure 5a

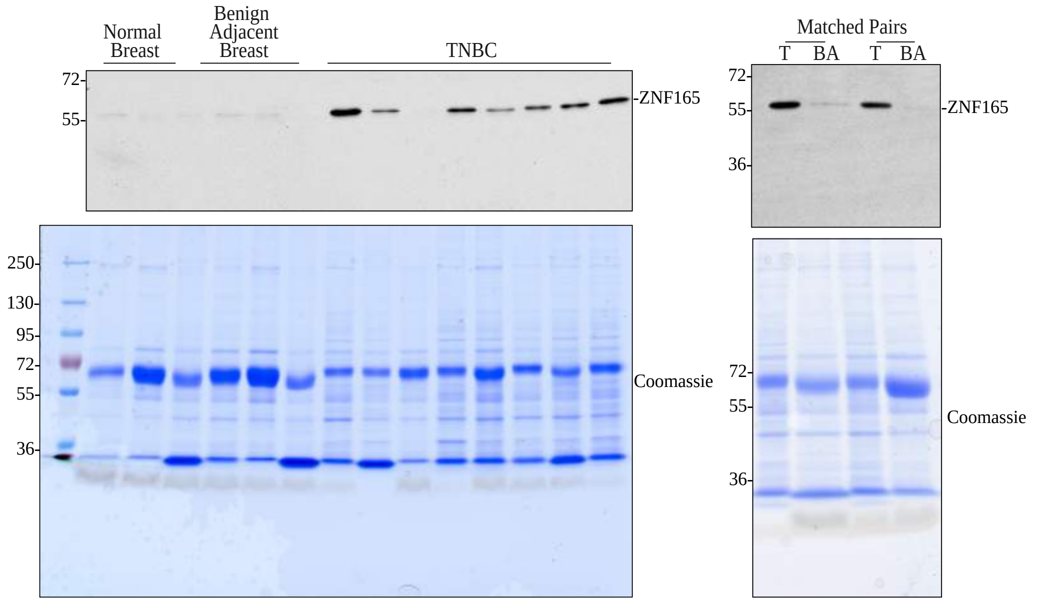

Figure 5f

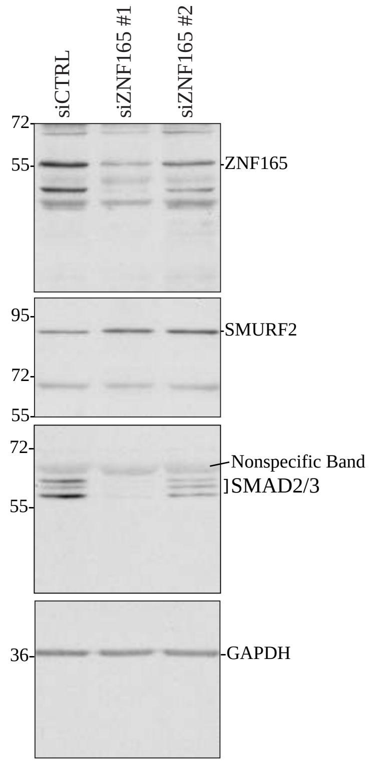

Figure 5g

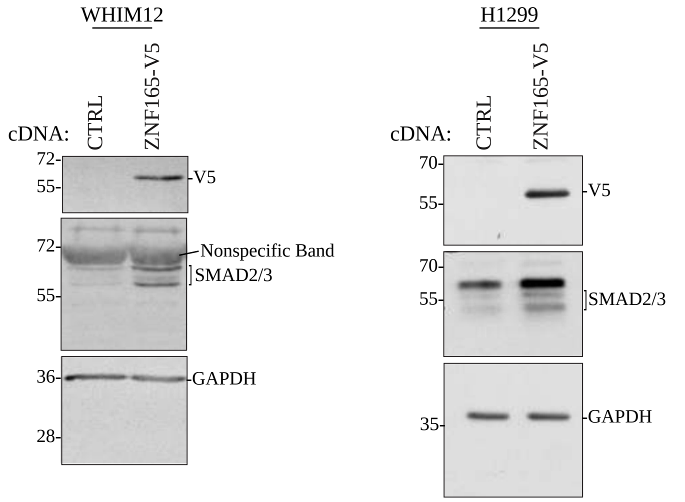

Figure 5j

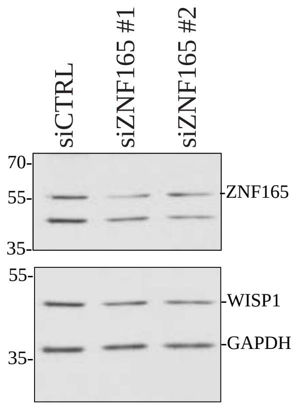

Figure 5k

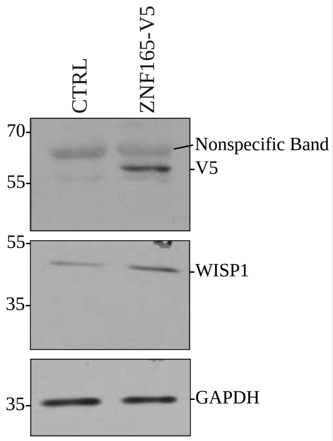

Supplementary Figure 7: Whole blots for Figure 5

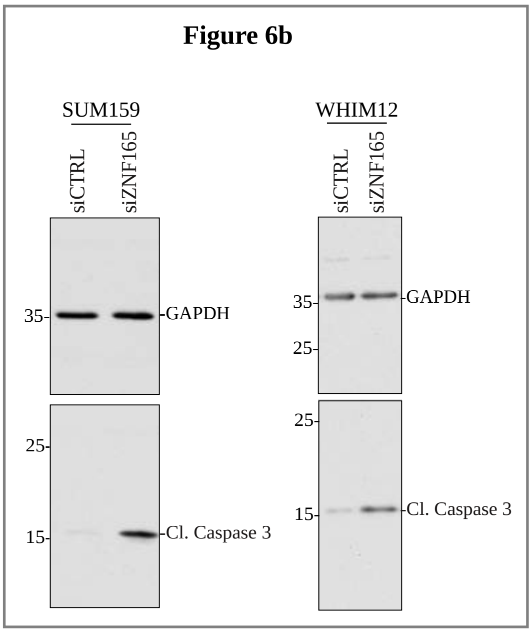

Supplementary Figure 8: Whole blots for Figure 6

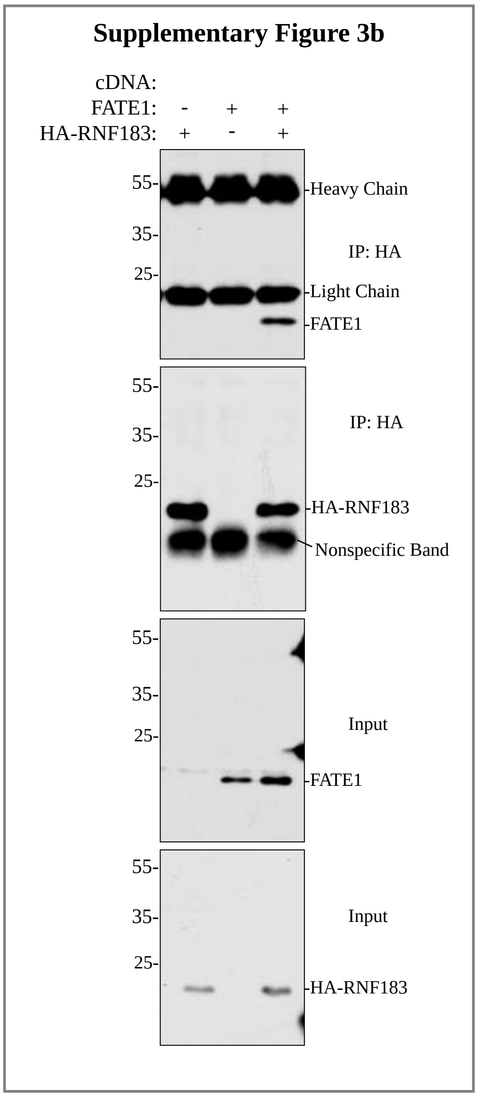

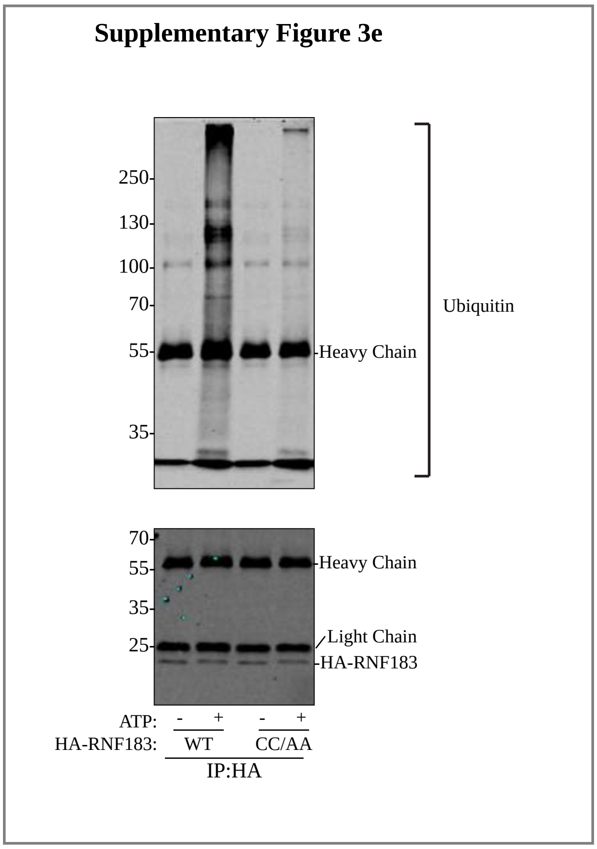

Supplementary Figure 9: Whole blots for Supplementary Figure 3

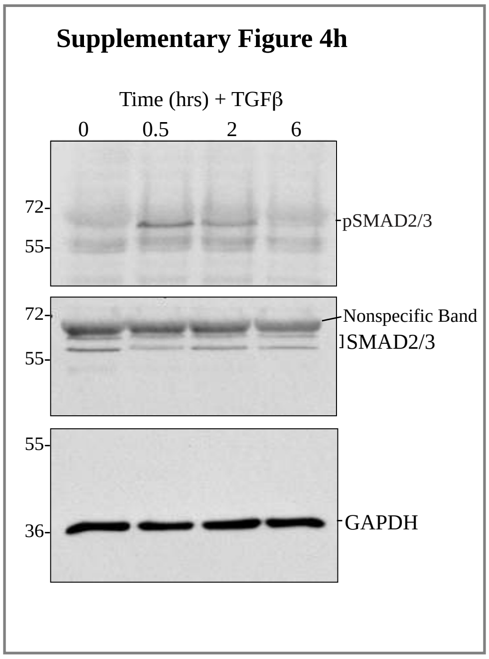

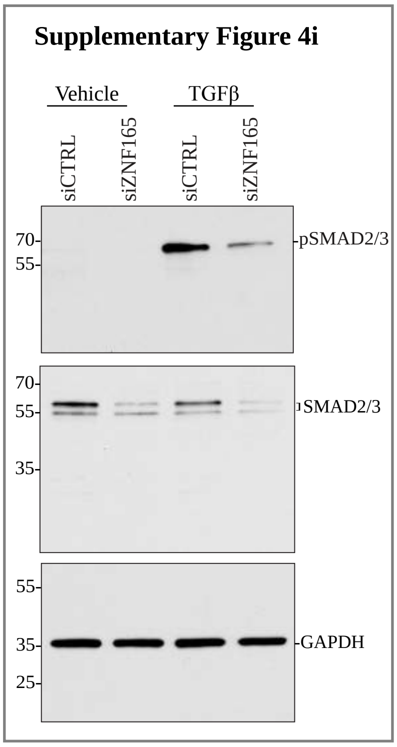

Supplementary Figure 10: Whole blots for Supplementary Figure 4

Supplementary References

1. Pei H, et al. FKBP51 affects cancer cell response to chemotherapy by negatively regulating Akt. Cancer cell **16**, 259-266 (2009).

2. Stearman RS, et al. Analysis of orthologous gene expression between human pulmonary adenocarcinoma and a carcinogen-induced murine model. The American journal of pathology **167**, 1763-1775 (2005).

3. Cancer Genome Atlas N. Comprehensive molecular portraits of human breast tumours. Nature **490**, 61-70 (2012).

4. Cancer Genome Atlas N. Comprehensive molecular characterization of human colon and rectal cancer. Nature **487**, 330-337 (2012).

5. Sorlie T, et al. Gene expression patterns of breast carcinomas distinguish tumor subclasses with clinical implications. Proc Natl Acad Sci U S A **98**, 10869-10874 (2001).

6. Wurmbach E, et al. Genome-wide molecular profiles of HCV-induced dysplasia and hepatocellular carcnoma. Hepatology **45**, 938-947 (2007).

7. Yoshihara K, et al. Gene expression profiling of advanced-stage serous ovarian cancers distinguishes novel subclasses and implicates ZEB2 in tumor progression and prognosis. Cancer science **100**, 1421-1428 (2009).

8. Biewenga P, et al. Gene expression in early stage cervical cancer. Gynecologic oncology **108**, 520-526 (2008).

9. Yu H, et al. Next-generation sequencing to generate interactome datasets. Nat Methods **8**, 478-480 (2011).

10. Rual JF, et al. Towards a proteome-scale map of the human protein-protein interaction network. Nature **437**, 1173-1178 (2005).
